# Pacing Strategies Differ by Sex and Rank in 2020 CrossFit^®^ Open Tests

**DOI:** 10.3390/sports11100199

**Published:** 2023-10-11

**Authors:** Gerald T. Mangine, Elisabeth K. Zeitz, Joshua D. Dexheimer, Ashley Hines, Brandon Lively, Brian M. Kliszczewicz

**Affiliations:** 1 Exercise Science, Kennesaw State University, Kennesaw, GA 30144, USA; ahines19@students.kennesaw.edu (A.H.); blivel12@students.kennesaw.edu (B.L.); bkliszcz@kennesaw.edu (B.M.K.); 2 Kinesiology, New Mexico State University, Las Cruces, NM 88003, USA; ezeitz@nmsu.edu; 3 Health Sciences, Liberty University, Lynchburg, VA 24515, USA; jdexheimer@liberty.edu

**Keywords:** fitness assessment, sport-specific, athlete classification, high-intensity functional training, sex differences

## Abstract

This retrospective study collected video recordings of a random selection of eighty men and women (*n* = 160) completing all five tests of the 2020 CrossFit^®^ Open. All competitors were ranked within the top 10,000 overall but were sub-divided based on whether they ranked within the top 10% of their respective divisions. To examine the effect of sex and rank on pacing strategy, video analysis quantified the overall repetition completion rate on each test, as well as per minute (or round) repetition completion rates for each test’s individual exercises, quantity of failed repetitions, break times, and transition times. All per minute (or round) data were aggregated into first- and last-half or total test average, slopes, and coefficient of variation. Sex and rank analyses of variance were performed on averages, slopes, and coefficients of variation for each variable calculated over the first and last halves of each test, except test 5 (total only). The top 10% of men were 17.5% faster (*p* < 0.001) than everyone else in tests 1, 3, and 5. The top 10% of women and remaining men were ~9.5% faster than remaining women in tests 1 and 3. In test 5, the remaining men were faster than top 10% of women (~11.2%, *p* < 0.001), and both were faster than the remaining women. In tests 2 and 4, the top 10% of athletes were 9.7% faster (*p* < 0.001) than remaining athletes, and at the same time, men were 7.7% faster (*p* < 0.001) than women. Analysis of each test’s components revealed the top 10% of competitors to be faster and more consistent in most areas, while men were generally faster than women in gymnastics components and more consistent with their pace for resistance training exercises. These data provide insight into the differential factors linked to success in the men’s and women’s CFO divisions.

## 1. Introduction

The CrossFit^®^ Open (CFO) is a multi-week, international fitness competition that serves as the preliminary qualifying stage of the CrossFit Games^TM^ [1]. Each week, athletes are tasked with completing one or more physical tests (i.e., workouts) that uniquely challenge a combination of their strength, endurance, and sport-specific skill [2,3]. Currently, competitors are given four days to complete each test and submit their best score to the competition submission portal [4]. Performances are verified either in-person by a judge or by competition officials via video submission, and then ranked. Assigned ranks serve as points-earned (e.g., rank #1 earns 1 point, rank #10 earns 10 points), and points accumulate over each week of the competition. After the CFO concludes, the current rules identify the top 10% of competitors (i.e., the lowest scoring 10%) within each sex division, and those athletes advance to the next stage of competition [3]. Although some CFO tests may be repeated in later competitions [5], most are unique and the details of any test are not known until the week of its individual release. Athletes who aim at earning a rank within the top 10% should not only work at developing the physiological traits that might impact success [6,7,8,9,10,11,12,13], but also their strategic approach to pacing a variety of possible test designs [14,15]. Since it is impossible to know the specific details of future CFO tests [16], trainees may find benchmark workouts to be useful for monitoring progress and predicting future CFO success. In addition to several existing “named” workouts, whose details have been standardized across training facilities, after a CFO test’s first appearance, it becomes a benchmark workout to be incorporated into normal training. To this end, a recent article by Mangine and colleagues [17] published normative scores for men and women in each CFO test assigned between 2011 and 2021. Trainees can use these scores to estimate how their current performance might have ranked in the associated year(s) that a specific test appeared in CFO programming. An interesting finding related to the secondary aim of that study was the performance differences noted between men and women in nearly every test.

Although men and women compete in separate divisions [3], most of the time they are assigned the same list of exercises to complete in each CFO test (55 out of 60 total tests from 2011 to 2021). Unlike teen athletes, masters athletes, and the actual “scaled” division, prescription in the “as prescribed” (i.e., Rx) division is also scaled (i.e., modified) between men and women for one or more exercises [2]. It might be presumed that this particular scaled prescription is meant to account for natural, physiological differences between sexes [18,19] and avoid drastic differences in test difficulty. However, some exercise types or modalities have never received scaled prescription, despite being tied to relevant (to sports performance) physiological attributes known to be different between sexes. In 91% of scaled tests, the programming component that was prescribed differently to men and women involved load assignments for weight-training exercises. Men are assigned heavier loads in an attempt to account for differences in strength capability [18]. Likewise, equating strength (or power) is a plausible reason for the scaling of non-weight-training exercises (in ~33% of tests), which are exclusively limited to assigned medicine ball weight, heights of targets, and boxes assigned for wall ball (WB) shots and box jumps (BJ), respectively. Greater strength in men might also be inferred as the reason for why gymnastics exercises are not scaled. Men are typically heavier than women [18,20], and would naturally require greater strength to maneuver their body about a pull-up bar or walk/push-up from a handstand position. In contrast, prescription for traditional cardiovascular modalities (usually rowing and jumping rope) has never differed between men and women in any CFO test [2].

CrossFit^®^-style workouts and CFO tests are commonly designed to encourage maximizing workout density [15]. When tests are scored by time-to-completion (TTC), they are best accomplished when the individual performs the assigned exercise repetitions as quickly as possible, efficiently transitions between exercises, and minimizes their autoregulated rest breaks. Minimizing transition time and breaks is even more important when tests ask competitors to complete ‘as many repetitions as possible’ (AMRAP) within an assigned duration, especially when there are physical limitations as to how quickly the individual exercises might be performed (e.g., the medicine ball cannot be made to drop faster from the target). The overall ability to maximize workout density within test durations lasting several minutes depends on the individual’s capacity to supply energy to exercising muscle and process deleterious metabolic byproducts [21,22], particularly when involving continuous effort movements (e.g., rowing and jumping rope). That is, CFO testing outcomes are affected by aerobic and anaerobic capacity [6,7,8,9,10,11,12], that are attributes often known to differ between men and women [19]. Thus, it was not surprising when two CrossFit^®^-style (non-CFO) workouts that scaled all exercises (i.e., weight-training loads and rowing) except for one (i.e., burpees) reported no sex differences [23]. Meanwhile, Mangine and colleagues [17] reported sex differences in 56 of the 60 CFO tests created between 2011 and 2022, with men significantly outperforming women in 41 tests (~68%). These widespread differences would suggest that the prescription was not appropriately scaled between men and women in most CFO tests. However, beyond that statement, there is little insight to be gained about sex differences in relation to scaled and unscaled workout components when the examination is limited to overall test performance. A more comprehensive understanding of the sex-based differences could only be made after CFO tests were broken down into their individual components (i.e., each exercise, transition, and break). Currently, only a pair of small-sample (<12 participants) studies have broken down a CrossFit^®^-style workout [11] or CFO tests [14] into individual components (i.e., exercises, transitions, breaks), and neither made comparisons between men and women. In fact, no study has compared the pacing strategies employed by men and women for each component of any CrossFit^®^-style workout, nor has any study ever made such comparisons between competitors who would and would not advance beyond the CFO. Therefore, the purpose of this investigation was to examine the effect of sex and rank on pacing strategies employed in individual CFO test components. The findings of this study would provide useful insight into the factors that might explain why men and women, as well as higher ranking competitors, score differently in CFO tests.

## 2. Materials and Methods

### 2.1. Experimental Design

Submitted video recordings of male and female athletes completing the five fitness tests programmed for the 2020 CFO competition were analyzed from the official competition leaderboard [4]. Recordings of the 2020 CFO performances were selected because they represented the most recent competition at the commencement of this study. Since the video recordings were pre-existing and publicly available, the University’s Institutional Review Board classified data collected from this source for research purposes as exempt and did not require athletes to provide their informed consent (IRB #16-215). The analysis of each test involved quantifying the duration and repetitions completed for each exercise effort, transition, break, and failed repetition. Due to differences in programming assigned to men and women [2,3], all repetition sets were converted into a rate (repetitions × s^−1^), while variations amongst competitors’ auto-regulated breaks and transition durations necessitated their conversion to respective averages. These values were either calculated per round (test 1) or per minute (tests 2–5) within each test. To observe differences in pacing strategy over the course of each test, the per round or per minute values were then collated over each test’s first and second halves, and comparisons were made between men and women who had ranked within and outside the top 10%.

### 2.2. Participants

The inclusion criteria for this study required all participants to have earned a rank within the top 10,000 of the men’s and women’s divisions during the 2020 CFO and to have submitted a video recording of themselves completing each of the five 2020 CFO tests to the online leaderboard [4]. Men (n = 855) and women (n = 416) who met these initial inclusion criteria were identified and placed in random order. Based on previous CFO performance comparisons between men and women [17,24], a priori analysis estimated a total sample of 128 participants would be needed to observe true differences via a two-tailed *t*-Test using the following thresholds (α = 0.05, β = 0.80, *d* = 0.50). Due to the amount of time required to analyze each athlete’s set of test recordings and the expectation that the process might reveal instances when an effort did not meet a test’s prescribed programming standards for individual men and women (i.e., Rx) [2,3], the sample target was increased to 160 participants (80 men and 80 women). This total would still provide sufficient statistical power at a 20% loss. After randomly ordering cases, the first 80 cases in each sex-division that met the initial inclusion criteria were selected and screened to ensure that the correct test was completed by the same person in all five video submissions associated with an athlete’s profile. If a specific case failed to meet this secondary criterion on any test, it was removed and replaced by the next available case on the list to maintain a total of 80 men and 80 women. This screening process identified 40 men and 114 women who did not meet secondary criteria. Following this screening process, the remaining 80 men and 80 women were retained for analysis, regardless of whether a specific test effort did not meet programming standards. That is, only data from that specific effort (not the entire case) were removed from the final statistical analysis. This final process removed 10 cases (men = 4, women = 6) from test 1, five cases (men = 2, women = 3) from test 2, eight cases (men = 4, women = 4) from test 3, nine cases (men = 6, women = 3) from test 4, and seven cases (men = 6, women = 1) from test 5.

For descriptive purposes, each athlete’s age, height (in cm), body mass (in kg), and CFO competitive history were also recorded from the profile linked to their position on the online leaderboard [4]. To examine the effect of rank on pacing strategy, the sample was sub-divided into athletes who ranked within the top 10% (men = 16; women = 47) of all Rx competitors in the 2020 CFO who met previously described criteria (men = 64; women = 33) [17]. Competition history included whether an athlete officially participated in a previous CFO and their associated overall rank. This information was further used to determine each athlete’s highest rank ever earned in a previous CFO, their rank in 2019 (when applicable), and the number of consecutive CFO appearances leading up to the 2020 CFO. The present sample included 18 athletes who participated in their first CFO in 2020, and another 10 whose first CFO was in 2019. Descriptive data about the present sample are presented in Table 1.

### 2.3. Competition Format and Test Analysis

The 2020 CFO competition occurred over five consecutive weeks beginning on 10 October 2019. On Thursday evening of each week, one test was released via live online broadcast, and competitors were given four days to complete the test at their normal training facility and upload their best score to the online leaderboard [4]. Each release would primarily focus on the instructions for completing the prescribed version for competitors in the main division (i.e., Rx), though modified instructions for all other competitive divisions (e.g., scaled, masters, teens, etc.) were also released online at this time. Though instances have existed where competitors from all divisions were tasked with completing the exact same test, modified versions typically program variants in Rx exercises, prescribe different repetition counts (per exercise), and/or prescribe different intensity loads [3]. Because these differences alter the assigned workload, equating drastically different tests is inherently difficult [15], and less non-Rx competitors submit video recordings of their efforts; therefore, only Rx performances were considered for this study. The programming details for each test and standardized methods for timing each exercise are described in Table 2, whereas descriptions of official movement standards for each exercise appearing in the 2020 CFO may be found elsewhere [2,3].

The analysis of each test involved recording start and end times for each exercise, transition between exercises, and break (defined as ceasing continuous movement within a set for longer than 2 s) using standardized procedures for each exercise (see Table 2). These data, as well as repetitions completed and failed, were entered into a spreadsheet (Microsoft Excel v. 365; Microsoft Corporation, Redmond, VA, USA) to calculate the time (in s) devoted to each exercise, transition, and break during each round (tests 1, 3, and 4) or minute (tests 2 and 5). Time devoted to an exercise and completed repetitions within a period (i.e., round or minute) were used to calculate repetition completion rate (repetitions × s^−1^), while each transition’s and break’s count and duration within that period were used to calculate average transition and average break (in s), respectively. Subsequently, the average, slope (per round or minute), standard deviation (SD), and coefficient of variation (%, SD divided by average) for each exercise rate, average transition, and average break were averaged across the first and last halves of each test, except for test 5 where these were calculated across the entire test. Total failed repetitions were counted for each exercise over each entire test. Failed repetitions encompassed repetitions that did not meet a movement’s official standards (verified by competition officials) [2,3] or when the count exceeded prescription on a given set or test.

### 2.4. Statistical Analysis

Three-way (sex-division (men, women) × rank (top 10%, remaining) × time (first half, last half)) analyses of variance (ANOVA) with repeated measures were performed on all pacing variables derived from tests 1–4. The assumption of normal distribution was verified via the Shapiro–Wilk test; however, due to the exploratory nature of this study and because sphericity tests cannot not be performed when repeated elements only consist of two levels, sphericity was assumed in all cases. Since test 5 allowed competitors to complete assigned programming in any order and could not be divided equally in half, pacing variables from it along with overall rank and test repetition completion rate were assessed via two-way (sex-division × rank) ANOVAs. Pairwise comparisons were performed following any significant F-ratio using the Bonferroni adjustment. Significance was accepted at an alpha level of *p* ≤ 0.05. Significant differences were further evaluated by effect sizes (eta-squared, η^2^) using the following thresholds: small (0.10–0.24), medium (0.25–0.39), and large (>0.40) [25]. All data are reported as mean ± SD. All statistical analyses were performed using JASP (v0.16.1; Amsterdam, the Netherlands).

## 3. Results

### 3.1. Overall Performance

**Figure 1:**
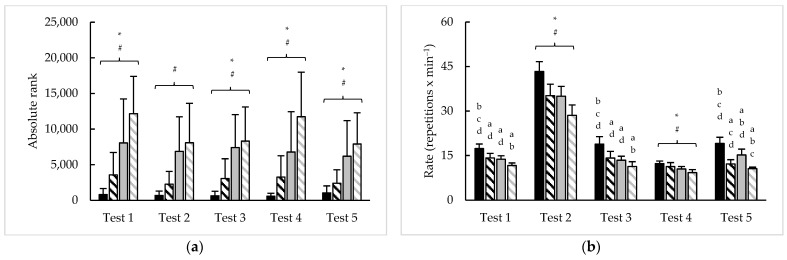
Sex and rank differences in (**a**) absolute rank and (**b**) repetition completion rate on each 2020 CFO test (mean ± SD). * = significant (*p* < 0.05) difference between sexes; # = significant (*p* < 0.05) difference between ranks; a–b = significantly (*p* < 0.05) different than (a. top 10% men (black bars); b. top 10% women (black striped bars); c. remaining men (grey bars); d. remaining women (grey striped bars)).

Sex and rank differences in overall performance in each 2020 CFO test are illustrated in Figure 1. Except test 2 (F = 3.5, *p* = 0.063, η^2^ = 0.02), significant main effects for sex were seen in absolute rank with all tests (F = 4.6–18.9; *p* < 0.05; η^2^ = 0.02–0.08). Of course, significant main effects for rank were observed in absolute rank with all tests (F = 55.8–77.8, *p* < 0.001, η^2^ = 0.27–0.33). Sex × rank interactions were seen for repetition completion rate in tests 1 (F = 4.8, *p* = 0.030, η^2^ = 0.01), test 3 (F = 14.0, *p* < 0.001, η^2^ = 0.04), and test 5 (F = 14.6, *p* < 0.001, η^2^ = 0.02), including the tie-break time for test 5 (F = 45.6, *p* < 0.001, η^2^ = 0.22). For tests 2 and 4, main effects for sex (F = 31.9–128.6, *p* < 0.001, η^2^ = 0.12–0.31) and rank (F = 94.6–135.0, *p* < 0.001, η^2^ = 0.32–0.35) were noted for repetition completion rate. 

### 3.2. Test 1 Component Pacing

**Figure 2:**
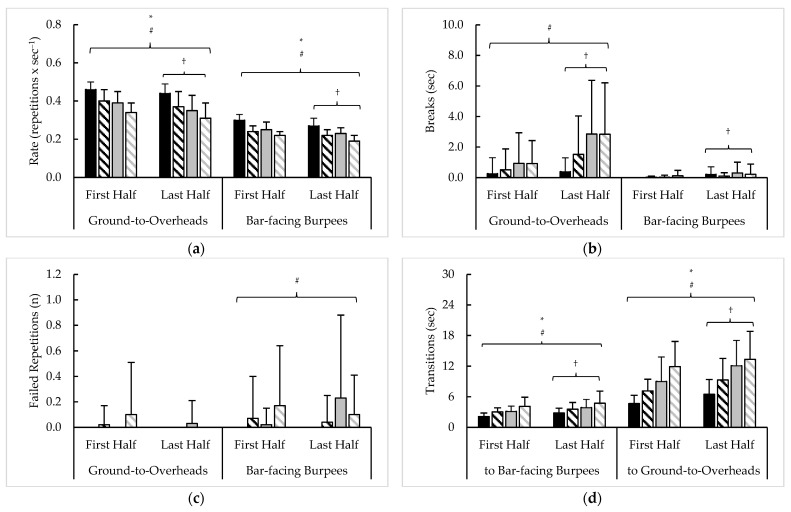
Test 1 sex and rank differences in average (**a**) repetition completion rate, (**b**) breaks, (**c**) failed repetitions, and (**d**) transitions between exercises. † = significant (*p* < 0.05) difference between halves; * = significant (*p* < 0.05) difference between sexes; # = significant (*p* < 0.05) difference between ranks; a–b = significantly (*p* < 0.05) different.

Pacing measures averaged across 10 rounds of test 1, as well as their variability, are presented in Figure 2 and Table 3, respectively. Time × rank interactions were seen for G2OH repetition completion rate (F = 4.7, *p* = 0.033, η^2^ < 0.01) and G2OH breaks (F = 10.8, *p* = 0.001, η^2^ = 0.01) along with a main effect for sex for repetition completion rate (F = 22.4, *p* < 0.001, η^2^ = 0.10). No differences were seen with failed repetitions. Main effects for time (F = 69.3, *p* < 0.001, η^2^ = 0.06), sex (F = 82.6, *p* < 0.001, η^2^ = 0.24), and rank (F = 49.7, *p* < 0.001, η^2^ = 0.14) were seen for BFB repetition completion rate, while only a main effect for time with BFB breaks (F = 11.1, *p* = 0.001, η^2^ = 0.03) and a main effect for rank with BFB failed (“extra”) repetitions (F = 4.7, *p* = 0.032, η^2^ = 0.02) were noted. Main effects for time (F = 42.3–44.9, *p* < 0.001, η^2^ = 0.03–0.04), sex (F = 10.3–12.7, *p* < 0.002, η^2^ = 0.04–0.06), and rank (F = 20.0–40.9, *p* < 0.001, η^2^ = 0.10–0.18) were also noted for transitions to G2OH and BFB. No other differences were seen.

Analysis of test 1 variability revealed main effects for time in G2OH repetition rate slope (F = 32.1, *p* < 0.001, η^2^ = 0.11) and CV (F = 4.5, *p* = 0.036, η^2^ = 0.01), as well as the slope of G2OH breaks (F = 6.4, *p* = 0.013, η^2^ = 0.03). Main effects for sex (F = 7.4–7.5, *p* = 0.007, η^2^ = 0.02–0.03) and rank (F = 6.8–14.8, *p* < 0.010, η^2^ = 0.02–0.05) were also noted for the CVs of G2OH repetition rate and breaks. Time × sex interactions were seen with BFB repetition rate slope and CV (F = 6.0–14.6, *p* < 0.05, η^2^ = 0.01–0.03), and main effects for time were noted with the slope and CV of BFB breaks (F = 6.5–10.4, *p* < 0.05, η^2^ = 0.03). Finally, whereas time × rank and time × sex interactions were noted with the slopes of transitions to BFB (F = 11.3–13.9, *p* = 0.001, η^2^ = 0.04–0.05) and G2OH (F = 11.2–19.8, *p* = 0.001, η^2^ = 0.03–0.05), only main effects for time (F = 7.6, *p* = 0.006, η^2^ = 0.02), sex (F = 7.9, *p* = 0.006, η^2^ = 0.03), and rank (F = 5.1, *p* = 0.025, η^2^ = 0.02) were seen with the CV of transitions to G2OH. No other differences were observed.

### 3.3. Test 2 Component Pacing

**Figure 3:**
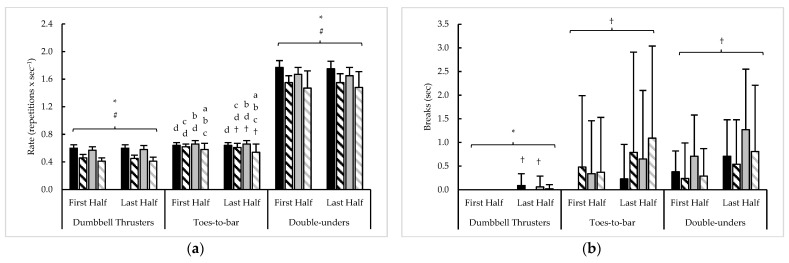

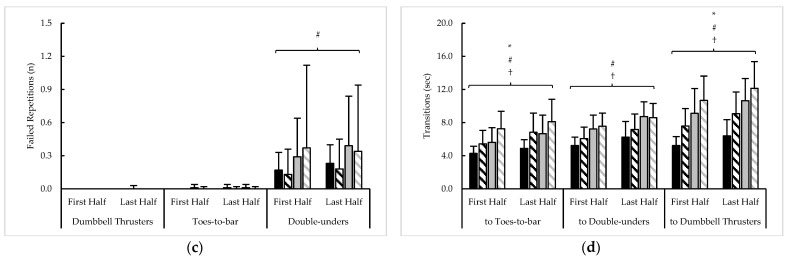
Test 2 sex and rank differences in average (**a**) repetition completion rate, (**b**) breaks, (**c**) failed repetitions, and (**d**) transitions between exercises. † = significant (*p* < 0.05) difference between halves; * = significant (*p* < 0.05) difference between sexes; # = significant (*p* < 0.05) difference between ranks; a–b = significantly (*p* < 0.05) different than (a. top 10% men (black bars); b. top 10% women (black striped bars); c. remaining men (grey bars); d. remaining women (grey striped bars)).

Pacing measures averaged across 20 min of test 2, as well as their variability, are presented in Figure 3 and Table 4, respectively. Main effects for sex (F = 61.1–286.0, *p* < 0.001, η^2^ = 0.25–0.60) and rank (F = 11.5–16.8, *p* < 0.001, η^2^ = 0.04–0.05) were seen with average DBT and DU repetition completion rates. During these two exercises, a time × sex interaction was seen with DBT breaks (F = 4.3, *p* = 0.040, η^2^ = 0.01) and a main effect for time with DU breaks (F = 32.2, *p* < 0.001, η^2^ = 0.04). With average TTB repetition rate, time × sex (F = 13.1, *p* < 0.001, η^2^ = 0.01), time × rank (F = 5.1, *p* = 0.025, η^2^ < 0.01), and sex × rank (F = 12.3, *p* = 0.001, η^2^ = 0.06) interactions were found, and only a main effect for time was seen for TTB breaks (F = 13.8, *p* < 0.001, η^2^ = 0.01). Of the three exercises, only a main effect for rank was seen in failed DU repetitions (F = 5.3, *p* = 0.023, η^2^ = 0.03); otherwise, failed repetitions were similar across competitors for DBT and TTB. Main effects for time (F = 44.2–51.3, *p* < 0.001, η^2^ = 0.04), sex (F = 20.2–21.4, *p* < 0.001, η^2^ = 0.08–0.09), and rank (F = 20.5–66.5, *p* < 0.001, η^2^ = 0.09–0.23) were noted when competitors transition to TTB and DBT, but main effects were limited to time (F = 97.6, *p* < 0.001, η^2^ = 0.07) and rank (F = 43.4, *p* < 0.001, η^2^ = 0.18) when transitioning to DU. No other differences were observed.

Analysis of test 2 variability revealed main effects for time (F = 7.5, *p* = 0.007, η^2^ = 0.02), sex (F = 28.0, *p* < 0.001, η^2^ = 0.08) and rank (F = 6.1, *p* = 0.015, η^2^ = 0.02) with the CV of DBT repetition rate, and a time × sex interaction (F = 4.1, *p* = 0.045, η^2^ = 0.01) for the CV of DBT breaks. A main effect for sex (F = 8.5, *p* = 0.004, η^2^ = 0.03) was seen with TTB rate slope, while main effects for time (F = 12.5, *p* = 0.001, η^2^ = 0.02), sex (F = 78.1, *p* < 0.001, η^2^ = 0.21), and rank (F = 11.4, *p* = 0.001, η^2^ = 0.03) were seen with the CV for TTB rate, along with a main effect for time with the CV for TTB breaks (F = 13.2, *p* < 0.001, η^2^ = 0.04). For DU, a sex × rank interaction (F = 4.6, *p* = 0.034, η^2^ = 0.01) was noted for the slope of DU rate, a main effect for rank (F = 4.9, *p* = 0.028, η^2^ = 0.01) with the slope of DU breaks, main effects for time (F = 7.3, *p* = 0.008, η^2^ = 0.01) and sex (F = 80.2, *p* < 0.001, η^2^ = 0.23) for the CVs of DU rate and breaks, along with a main effect for rank with the CV of DU rate (F = 21.4, *p* < 0.001, η^2^ = 0.06). Time × sex interactions were found with the slope of transitions between all three exercises (F = 4.0–12.5, *p* < 0.05, η^2^ = 0.01–0.03), along with time × rank interactions with the slope of transitions to TTB (F = 6.7, *p* = 0.011, η^2^ = 0.02) and DBT (F = 11.3, *p* = 0.001, η^2^ = 0.03). A sex × rank × time interaction was found for the CV of DU transitions (F = 10.8, *p* = 0.001, η^2^ = 0.02), and then main effects for time (F = 12.5–15.5, *p* = 0.001, η^2^ = 0.02–0.03), sex (F = 43.0–54.1, *p* < 0.001, η^2^ = 0.13–0.16), and rank (F = 25.4–36.5, *p* = 0.001, η^2^ = 0.08–0.11) for CVs of TTB and DBT transitions. No other differences were seen.

### 3.4. Test 3 Component Pacing

**Figure 4:**
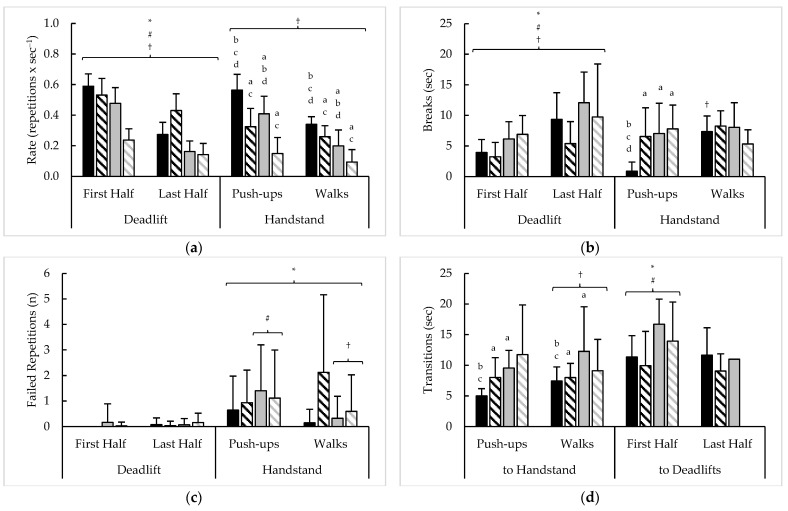
Test 3 sex and rank differences in average (**a**) repetition completion rate, (**b**) breaks, (**c**) failed repetitions, and (**d**) transitions between exercises. † = Significant (*p* < 0.05) difference between halves; * = Significant (*p* < 0.05) difference between sexes; # = Significant (*p* < 0.05) difference between ranks; a–b = Significantly (*p* < 0.05) different than (a. top 10% men (black bars); b. top 10% women (black striped bars); c. remaining men (grey bars); d. remaining women (grey striped bars)).

Pacing measures averaged across six rounds of test 3, as well as their variability, are presented in Figure 4 and Table 5, respectively. Main effects for time (F = 72.7–899.8, *p* < 0.001, η^2^ = 0.18–0.63), sex (F = 6.7–8.5, *p* < 0.01, η^2^ = 0.01–0.02), and rank (F = 17.0–57.2, *p* < 0.001, η^2^ = 0.05–0.07) were observed for average DL repetition rate and breaks with no differences amongst competitors with failed DL repetitions. A sex × rank interaction (F = 9.2, *p* = 0.003, η^2^ = 0.05) and main effect for time (F = 9.4, *p* = 0.003, η^2^ = 0.03) was noted with average transitions to HSPU-HSW. Then, sex × rank interactions were seen for the HSPU-HSW repetition rate (F = 4.4, *p* = 0.038, η^2^ = 0.01) and breaks (F = 7.1, *p* = 0.009, η^2^ = 0.03), along with a main effect for time for HSPU-HSW rate (F = 179.4, *p* < 0.001, η^2^ = 0.26), and time × rank (F = 12.9, *p* = 0.001, η^2^ = 0.05), and time × sex (F = 11.7, *p* = 0.001, η^2^ = 0.05) interactions for HSPU-HSW breaks. A time × rank interaction (F = 5.9, *p* = 0.016, η^2^ = 0.02) and main effect for sex (F = 7.0, *p* = 0.009, η^2^ = 0.02) was seen with HSPU-HSW failed repetitions, while main effects for sex (F = 4.5, *p* = 0.035, η^2^ = 0.03) and rank (F = 22.4, *p* < 0.001, η^2^ = 0.13) were noted with average transitions to DL. An insufficient number of competitors advanced to the fifth round of this test (top 10% men = 13, top 10% women = 7, remaining men = 2, remaining women = 0) and prevented comparisons involving transitions to DL in the last half of test 3. 

Analysis of test 3 variability revealed a time × rank interaction for the CV of DL repetition rate (F = 13.0, *p* < 0.001, η^2^ = 0.04) and main effects for time with the slopes and CVs of DL rate (F = 6.4–22.8, *p* < 0.05, η^2^ = 0.02–0.14) and DL breaks (F = 21.1–37.4, *p* < 0.001, η^2^ = 0.13–0.14), and a main effect for rank was seen with the CV of DL breaks (F = 4.6, *p* = 0.033, η^2^ = 0.02). Main effects for rank were also seen with the slope and CV of transitions to HSPU-HSW (F = 4.8–5.7, *p* < 0.05, η^2^ = 0.02–0.03). Then a time × rank interaction for the CV (F = 4.6, *p* = 0.034, η^2^ = 0.02) and main sex effect for the slope (F = 8.3, *p* = 0.005, η^2^ = 0.05) of HSPU-HSW rate were noted, along with a main time effect in the CV of HSPU-HSW breaks (F = 11.4, *p* = 0.001, η^2^ = 0.05). Main effects for sex were also seen for the slope and CV (F = 9.2–14.9, *p* < 0.10, η^2^ = 0.06–0.08) along with a main rank effect with the CV (F = 28.4, *p* < 0.001, η^2^ = 0.15) of transitions to DL.

### 3.5. Test 4 Component Pacing

**Figure 5:**
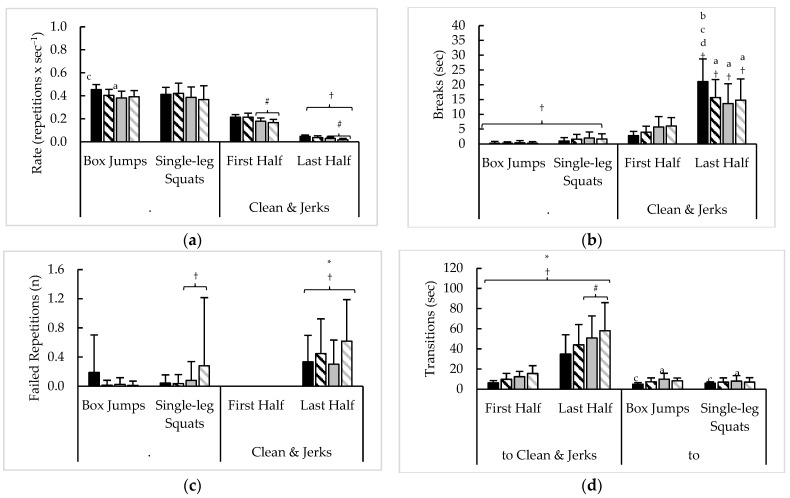
Test 4 sex and rank differences in average (**a**) repetition completion rate, (**b**) breaks, (**c**) failed repetitions, and (**d**) transitions between exercises. † = Significant (*p* < 0.05) difference between halves; * = Significant (*p* < 0.05) difference between sexes; # = Significant (*p* < 0.05) difference between ranks; a–b = Significantly (*p* < 0.05) different than (a. top 10% men (black bars); b. top 10% women (black striped bars); c. remaining men (grey bars); d. remaining women (grey striped bars)).

Pacing measures averaged across six rounds of test 4, as well as their variability, are presented in Figure 5 and Table 6, respectively. Analysis of averaged pacing across six rounds of test 4 revealed sex × rank × time interaction for average BJ-SLSQ repetition completion rate (F = 5.0, *p* = 0.027, η^2^ = 0.02), a main time effect for breaks (F = 66.3, *p* < 0.001, η^2^ = 0.16), and a time × rank interaction for failed BJ-SLSQ repetitions (F = 6.3, *p* = 0.013, η^2^ = 0.02). A time × rank interaction (F = 4.7, *p* = 0.032, η^2^ = 0.01) and main sex effect (F = 7.3, *p* = 0.008, η^2^ = 0.01) were then seen with transitions to CNJ. For CNJ, a time × rank interaction (F = 20.4, *p* < 0.001, η^2^ < 0.01) and main sex effect (F = 6.0, *p* = 0.015, η^2^ < 0.01) were seen with repetition rate, while a sex × time × rank (F = 7.1, *p* = 0.009, η^2^ = 0.01) and sex × time (F = 7.4, *p* = 0.007, η^2^ = 0.02) interactions were observed for breaks and failed repetitions, respectively. A sex × rank interaction (F = 4.1, *p* = 0.044, η^2^ = 0.02) was noted for transitions to BJ-SLSQ.

Analysis of test 4 variability revealed a time × rank interaction with the CV (F = 8.1, *p* = 0.005, η^2^ = 0.02) and main effects for time (F = 98.5, *p* < 0.001, η^2^ = 0.25) and sex (F = 4.5, *p* = 0.036, η^2^ = 0.01) with the slope of BJ-SLSQ rate. Time × sex (F = 4.9, *p* = 0.029, η^2^ = 0.01) and time × rank (F = 18.3, *p* < 0.001, η^2^ = 0.05) interactions were then noted for the slope of BJ-SLSQ breaks, but only a main time effect for the CV (F = 70.9, *p* < 0.001, η^2^ = 0.19). When transitioning to CNJ, a sex × rank × time interaction with the CV (F = 6.9, *p* = 0.009, η^2^ = 0.01) and a main time effect with slope (F = 27.3, *p* < 0.001, η^2^ = 0.10) were seen. A main time effect was observed for the slope of CNJ rate (F = 438.6, *p* < 0.001, η^2^ = 0.65), while time × sex (F = 17.0, *p* < 0.001, η^2^ = 0.04), time × rank (F = 80.4, *p* < 0.001, η^2^ = 0.18), and sex × rank (F = 5.6, *p* = 0.020, η^2^ = 0.01) interactions were observed with the CV. For CNJ breaks, a sex × rank × time interaction (F = 10.4, *p* = 0.002, η^2^ = 0.02) and time × rank interaction (F = 18.5, *p* < 0.001, η^2^ = 0.05) were noted for slope and CV, respectively. A main sex effect was also seen for the CV of CNJ breaks (F = 5.5, *p* = 0.020, η^2^ = 0.01). Time × rank (F = 9.2, *p* = 0.003, η^2^ = 0.02) and sex × rank (F = 8.5, *p* = 0.004, η^2^ = 0.02) interactions were found with the CV of transitions to BJ-SLSQ, along with a main time effect for slope (F = 9.8, *p* = 0.003, η^2^ = 0.07).

### 3.6. Test 5 Component Pacing

**Figure 6:**
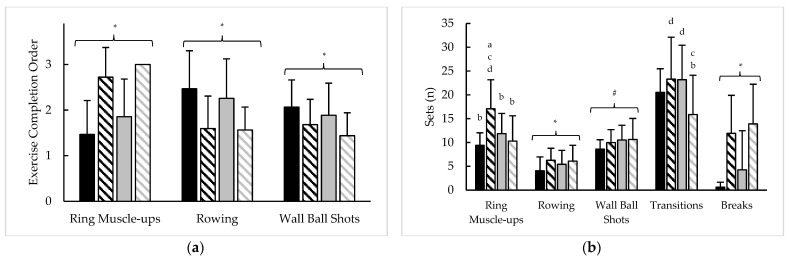

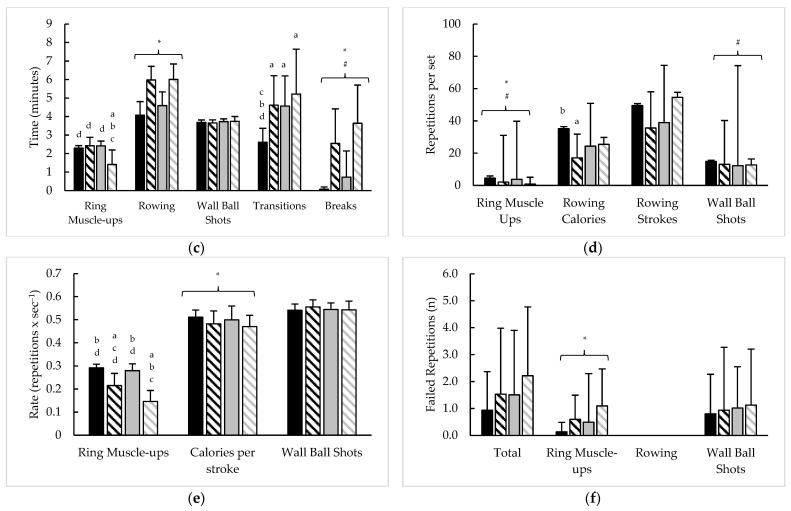
Test 5 sex and rank differences in average (**a**) exercise completion order, (**b**) sets devoted to each component, (**c**) time devoted to each component, (**d**) repetitions per set, (**e**) repetition completion rate, and (**f**) failed repetitions. * = significant (*p* < 0.05) difference between sexes; # = significant (*p* < 0.05) difference between ranks; a–b = significantly (*p* < 0.05) different than (a. top 10% men (black bars); b. top 10% women (black striped bars); c. remaining men (grey bars); d. remaining women (grey striped bars)).

Pacing measures averaged throughout test 5, as well as their variability, are presented in Figure 6 and Table 7, respectively. Analysis of averaged pacing strategy revealed sex × rank interactions for the number of sets (F = 25.1, *p* < 0.001, η^2^ = 0.13) and time (F = 41.6, *p* < 0.001, η^2^ = 0.17) devoted to RMU and RMU repetition completion rate (F = 13.5, *p* < 0.001, η^2^ = 0.04). Sex × rank interactions were also noted for rowing calories completed per set (F = 5.5, *p* = 0.020, η^2^ = 0.04), rowing strokes completed per set (F = 3.9, *p* = 0.049, η^2^ = 0.03), transitions performed (F = 12.2, *p* = 0.001, η^2^ = 0.07), and total time devoted to transitions (F = 4.4, *p* = 0.038, η^2^ = 0.02). Main effects for sex were observed for the order of exercise completion (F = 13.7–92.9, *p* < 0.001, η^2^ = 0.08–0.38), the number of sets and time devoted to rowing (F = 7.1–139.3, *p* < 0.010, η^2^ = 0.05–0.47), total breaks taken (F = 53.3, *p* < 0.001, η^2^ = 0.26), total break time (F = 77.5, *p* < 0.001, η^2^ = 0.33), RMU repetitions per set (F = 155.2, *p* < 0.001, η^2^ = 0.47), rowing SPM (F = 8.1, *p* = 0.005, η^2^ = 0.05), rowing rate (F = 8.2, *p* = 0.005, η^2^ = 0.05), and failed RMU repetitions (F = 4.3, *p* = 0.039, η^2^ = 0.03). Main effects for rank were seen with the number of WBS sets (F = 4.6, *p* = 0.033, η^2^ = 0.03), total break time (F = 8.1, *p* = 0.005, η^2^ = 0.04), RMU repetitions per set (F = 22.8, *p* < 0.001, η^2^ = 0.07), and WBS repetitions per set (F = 4.7, *p* = 0.031, η^2^ = 0.03).

Analysis of test 5 variability revealed main effects for sex (F = 28.4, *p* < 0.001, η^2^ = 0.16) and rank (F = 8.3, *p* = 0.005, η^2^ = 0.05) for the CV of RMU rate, slope, and CV of RMU breaks (F = 4.5–35.1, *p* < 0.05, η^2^ = 0.02–0.24), and CV of RMU break time (F = 7.4–30.9, *p* < 0.010, η^2^ = 0.06–0.24), as well as a sex × rank interaction for the slope of RMU break time (F = 5.0, *p* = 0.027, η^2^ = 0.02). A sex × time interaction was also noted for the CV of rowing repetition rate (i.e., calories per stroke per second) (F = 9.1, *p* = 0.003, η^2^ = 0.06). Main sex effects were observed for the slopes and CVs of WBS breaks (F = 5.7–9.5, *p* < 0.05, η^2^ = 0.04–0.06) and break time (F = 7.1–8.3, *p* < 0.010, η^2^ = 0.04–0.05), and a main rank effect was seen with WBS break time slope (F = 4.2, *p* = 0.042, η^2^ = 0.03). Sex × rank interactions were found with the CV of transitions (F = 5.2, *p* = 0.024, η^2^ = 0.03), and slope and CV of transition time (F = 4.0–24.5, *p* < 0.05, η^2^ = 0.02–0.13). No other differences were seen.

## 4. Discussion

The purpose of this study was to examine sex and rank differences in pacing strategies employed by Rx competitors of the 2020 CFO. To observe differences, recorded efforts in each of the five tests programmed that year were collected from competitors who ranked within the top 10,000 places of the men’s and women’s divisions. The athletes were further sub-divided by whether they had earned an overall rank within the top 10% of all competitors within their respective sex-division in 2020. Comparisons were then made across sex divisions, ranks (i.e., top 10% and remaining), and test halves (except test 5) to assess differences in overall pace, repetition completion rate for individual exercises, the use of breaks, transition efficiency, failed repetitions, and how each of these varied across the duration of exercise. As expected, top 10% competitors generally outpaced remaining competitors in each test and within the top 10%, men outpaced women in three of the five tests. Interestingly, the remaining men (i.e., those who did not place inside the top 10%) completed four tests just as fast as the top 10% women, and exceeded their pace in the fifth test (test 5). Analysis of test components provided further insight into which test aspects were advantageous to competitor classifications. Men (in general) and the top 10% of competitors (men and women) were usually faster in completing repetitions in approximately 60% of all prescribed exercises, and their pace varied less in approximately 40% of exercises. The top 10% of competitors more consistently transitioned between exercises nearly 80% of the time, while taking more consistent breaks in about half of the tests. Men and the top 10% of competitors were particularly faster in transitioning during tests 1 and 2. Among the classifications, the clearest distinctions were seen with gymnastics pacing followed by pacing when performing resistance training exercises with higher relative loads. These data greatly expand on a previous pilot study of ten 2016 CFO competitors [14], and is the first study to examine the effect of sex and rank on pacing strategy in discontinuous, multi-modal exercise.

The 2020 CFO featured three tests that required a high-volume of gymnastics exercises to be performed [2]. The competitors in this study repeated a set of six TTB repetitions an average of 20 times within a 20-min time limit (120 total repetitions) on test 2. To finish test 3, competitors had to complete 45 HSPU repetitions and traverse 150 feet while walking on their hands, and test 5 required 40 RMUs. The present study found that the top 10% men (and men in general) more quickly transitioned to these exercises and completed repetitions at a faster rate than all other competitors. In contrast, the remaining women were slowest in these or often failed to even perform or complete the assigned gymnastic work. In fact, nearly 80% of the remaining women failed to complete a single HSW repetition (i.e., walk five feet), whereas more than 70% of all other athletes in this study accomplished this in test 3. In test 5, the remaining women only averaged 10.7 RMU repetitions, while the top 10% women averaged 30.6, the remaining men averaged 39.5, and the top 10% men averaged 40 repetitions. Moreover, while men typically completed all RMU repetitions before completing any other test 5 exercise, women almost always completed them last and required approximately three more sets in total. These findings support recent observations made by Mangine and colleagues [5,17]. While calculating normative scores for all CFO tests from 2011 to 2021, *moderate*-to-*large* performance differences were noted to be in favor of men for nine of the thirteen CFO tests that required high-volume gymnastics to be completed within a 10–20-min duration [17]. In a follow-up study, men more consistently outperformed women whenever a CFO test involving a high volume of gymnastics was officially repeated, this in spite of athletes having an average of 2.4 years to improve their performance from the previous iteration [5]. The most obvious explanation for this is that men typically possess more upper-body strength endurance than women [18,19], and CFO gymnastics prescription has always been exactly the same for Rx competitors in both sex divisions [2]. That is, although physiological capability differences are expected, the men’s and women’s division competitors have always been prescribed the same amount of gymnastic work. One might argue that the gymnastic prescription is not the same because body mass is usually higher in men [18,20]. However, if that expected difference was sufficient to equate with work, then men and women should have been able to complete a similar number of repetitions in these exercises and at a similar rate.

The same expectation might be assumed to be true for tests involving resistance training movements (tests 1–4). Unlike gymnastics, resistance training loads are customarily different for competitors in the men’s and women’s divisions [2]; presumably, to account for known strength differences [18]. Since these differences typically cease to exist when loads are made relative to body mass [26,27,28], a reasonable hypothesis expects men and women to be capable of completing repetitions at a similar pace when using adequately scaled loads. Nevertheless, of all the comparisons made in this study, sex differences favoring men in resistance training movements were most expected. This was because CFO loads are not prescribed relative to body mass, but rather, they are apparently based on an estimated percent difference in strength [2]. Previously, Mangine and colleagues [17] noted faster completion rates by men in 65% of CFO tests that incorporated a resistance training exercise. Men were faster particularly when the test assigned higher relative loads to women (68.3 ± 2.7% of loads assigned to men), and slower than women when the test assigned lesser relative loads (64.7 ± 4.0% of loads assigned to men). Those observations were supported by our results. In tests 1 and 2, loads assigned to women were 68–70% of those assigned to men, and regardless of rank, men outperformed women in nearly every aspect of those tests. Likewise, test 3 paired higher relative DL loads to women (65.1–68.9% of loads assigned to men) with the previously discussed gymnastics and men more quickly transitioned to DL (first half only) and performed repetitions at a faster rate. Though it remains unclear how the difficulties women had with gymnastics affected their DL repetition completion rate, it may be surmised that the combination of the two impacted their capability of progressing through the last half of test 3, and in turn, the resultant metrics of variability (i.e., slope and CV) examined in this study. Indeed, approximately 50% of the top 10% women and remaining men completed 4–7 DL repetitions in round 5 and less than 1% of the top 10% men failed to complete a DL repetition in the final round. Conversely, the remaining women only averaged 12 of 21 repetitions in round 4, and then 94% and 100% failed to perform a single DL repetition in rounds 5 and 6. The absence of their data in later rounds would have led to a more weighted contribution from round 4 data when calculating variables over the test’s second half; thus, would be representative of comparatively less work. While a similar pattern could also be observed with CNJ repetitions across rounds 4–6 of test 4, a test that paired lower relative CNJ loads (64.5 ± 2.2% of the loads assigned to men) with partially scaled calisthenics (BJs and SLSQs), no sex differences were seen in repetition completion rate. Instead, the only relevant differences that might explain why men generally completed this test faster had to do with first-half consistency, their speed in transitions, and fewer failed repetitions over the last half of the test. The outcomes observed in the later halves of tests 3 and 4 should be viewed with caution.

Given how men generally outpaced women, the differences between the top 10% women and the remaining men are interesting to explore for the purposes of answering the hypothetical question of whether either could excel in the other’s sex-division. Although no differences were seen between these two groups in overall performance during the first four tests, the remaining men outpaced the top 10% women in test 5 by approximately 11.2%. Additionally, examination of specific test components showed that these men completed TTB, HSPU, and RMU repetitions at faster rates, and were more consistent in their CNJ rate, whereas top 10% women were faster in performing HSW repetitions. No other specific differences were seen and visual inspection of these sub-groups’ means when a main effect for sex was noted only implied an advantage for men in test 5. No other clear pattern of advantages was seen for either of these two sub-groups in tests 1–4. When contextualizing these findings, it is important to remember that the competitors selected for inclusion in this study ranked within the top 10,000 places of their respective divisions. After applying previously described criteria for Rx competitors [17], that placement threshold in men could more accurately be described as the point that approximately distinguished their top 20%. These sub-group comparisons suggest that 2020 CFO performances by the top 10% women were similar, with a few exceptions, to those of men who ranked between the top 10 and 20% of their division. This difference is consistent with previously reported scoring differences between a women’s division top 10% score in any CFO test (2011–2021) and where that score would have ranked amongst men [17]. Not counting maximal strength tests, a women’s division top 10% score for any test would, on average, place them 7.4% lower in the men’s division (or 6.6% lower for tests programmed between 2016 and 2021). Conversely, a top 10% score for men was, on average, similar to a top 2% score in women. The underlying reasons for this are still unclear but the findings of this study implicate gymnastics and assigned resistance training loads as the most likely factors.

Having a better understanding of why men more commonly outperform women in CFO tests is probably more important for competitive team events and training program design than it is for individual CFO competitors. This is because, currently, men and women compete in separate divisions and their respective performances have no impact on the other’s rankings [3]. Conversely, the disparities seen between the top 10% and remaining competitors helps to provide insight into the pacing strategies of those who ultimately advance beyond the CFO. In tests 1 and 2, the top 10% of competitors uniformly outperformed the remaining competitors within their respective sex-division in nearly every facet of these two tests. They consistently completed exercise repetitions at a faster rate over the entire workout, committed less failed (or extra) repetitions, took shorter breaks, and transitioned more quickly between exercises. Higher ranking competitors were more consistent in completing HSPU/HSW (test 3, men only) and RMU repetitions (test 5, women only) at faster rates. These observations support the idea that performances in CrossFit^®^-style workouts are determined by one’s capability of maintaining a faster and more consistent pace for the duration of exercise [14,15]. Though multiple areas of fitness have been found to predict performance [6,7,8,9,10,11,12,13], it would seem that for the 2020 CFO, skill and stamina in these particular aspects best distinguished performance between the top 10% and remaining competitors.

There were, however, instances when top 10% competitors moved faster but were less consistent. The top 10% women completed TTB repetitions faster than the remaining women, but the remaining competitors (men and women) generally kept a more consistent pace. Higher-ranking competitors were also faster but less consistent in DL repetition rates and transitions to both DL and HSPU/HSW (in men only) during test 3, as well as in CNJ repetition rates and transitions to CNJ and to SLSQ in test 4. Additionally, shorter test 5 breaks were seen in top 10% competitors but with inconsistencies amongst individual exercises. RMU breaks and break time were more variable while WBS breaks became progressively shorter in top 10% competitors. One explanation for each of these outcomes may be related to the unequal amount of work completed amongst participants. Scoring well in CFO tests is usually dependent on the number of repetitions completed within a given time limit, or completing all assigned work more quickly and/or before time runs out [2,3], and this inexorably leads to more work being completed by higher ranking competitors. For instance, within the 20-min time limit of test 2, the top 10% women completed approximately four extra rounds (~22 more TTB repetitions) compared to remaining women, and those additional repetitions would have factored into calculated averages, slopes, and CVs. The same can be said about the large percentage of remaining women who failed to reach rounds five and six of tests 3 and 4. Likewise, only two-thirds of remaining women completed more than two RMU repetitions. With fewer existing data points, calculated averages would be more heavily weighted towards repetitions completed when athletes were less fatigued, while calculated measures of variability would have been disproportionately low compared to those calculated for the top 10% of competitors. Although this explanation suggests that some of the observed variability differences should be viewed with skepticism, it is possible that competitors who are capable of averaging a significantly faster pace over the course of a test can afford to be less consistent. Indeed, not counting the extremely low CVs seen in remaining women during the last halves of tests 3 and 4, higher-ranking competitors were found to be 5–30% faster than remaining competitors across all these instances but fractionally less consistent. Based on this, one might hypothesize that there is a limit to how much speed should be sacrificed for the sake of a more consistent pace.

When reviewing these findings, it is important to maintain perspective and consider them within the context of this study’s inherent limitations. For instance, included competitors might be viewed as a specific sub-group within the top 10,000 athletes. This is because only those who submitted video recordings of their best attempt to the online leaderboard [4] for all five tests were considered for inclusion; a requirement that eliminated more than 90% of men and 95% of women who met our rank criteria. The decision to only consider the top 10,000 athletes within each division was made simply because video submissions became more scarce beyond this point, and recorded efforts were obviously necessary for video analysis to be possible. The secondary decision to only include competitors who submitted recordings for all five tests was made to ensure that different group compositions could not be a confounding factor when collating results across all test comparisons. While group compositions still varied for each test because individual efforts were excluded for not meeting a test’s programming and/or movement standards, these instances were relatively few (<5% of cases across all tests). The video submission requirement also provided evidence that the authenticity of the effort had been certified by competition officials [3], though it is still questionable as to whether each submission was critically examined by said officials. As noted above, cases still needed to be removed from analyses for reasons such as miscounted repetitions and incomplete efforts. It is also possible that the present sample was not representative of all athletes within the top 10,000 who recorded their efforts on all five tests. In lieu of video submission, competitors had the option of completing tests at a CrossFit^®^ affiliate in front of a certified judge who then submits the scoresheet for certified effort [3]. Possessing a video recording of the effort is still considered a best practice in case the validity of an attempt is questioned, but these may be submitted discreetly to competition officials. Regardless, the true number of competitors who actually recorded their efforts on all five tests but did not submit the video cannot be estimated, nor can their reasons for doing so be known. Another important limitation to mention involved the manner in which competitors were grouped by rank. This study used previously described criteria to identify a competitor’s effort as a valid attempt using Rx standards [17], and these criteria are more stringent than those required to earn an Rx rank in the CFO [2,3]. Briefly, the study criteria were designed to exclude attempts where it was apparent that the competitor intentionally performed only a limited number of repetitions for the entire test solely for the purpose of earning an Rx rank. These criteria reduced the overall pool of competitors and thus, affected percent rank calculations. Consequently, top 10% competitors defined in this study may actually be representative of a more exclusive group (i.e., higher ranking) than their stated rank implies. Nevertheless, these inclusion criteria were necessary to ensure that legitimate and complete efforts were being used for all comparisons, and each effort was as consistent as possible with those of similarly ranked athletes. Any missing data from an effort (e.g., athletes who did not attempt repetitions in rounds five and six of tests 3 and 4) were missing because it was a common occurrence amongst athletes of similar rank.

## 5. Conclusions

This is the first study to examine the effect of sex and rank on overall CrossFit^®^ (also referred to as high-intensity functional training) test pacing strategy, as well as on strategies employed for the individual components within each test. Although limited to the five tests of the 2020 CFO, our data suggest that strategies are different among men’s and women’s division competitors who ranked within and outside the top 10% of their respective divisions. As expected, the top 10% competitors produced better overall test scores but were also found to complete most individual aspects of each test at a faster pace and were more consistent in doing so than the remaining competitors. Likewise, men performed better than women in several areas, though the clearest distinctions were present when the test contained a gymnastics component. Both the top 10% men and remaining men performed better than all women in these types of exercises, except for handstand walking, where the top 10% women outpaced the remaining men. Otherwise, and except for test 5, the top 10% women and remaining men were comparable in most other aspects of each test. Conversely, the top 10% men outperformed other competitors in multiple areas, while the remaining women were least proficient among the top 10,000 athletes. These data are useful for a variety of practical and theoretical purposes. For those athletes who wish to advance in CFO ranking, these data provide evidence about which performance aspects distinguish rank and can help them identify areas they should work to improve and/or maintain. From a theoretical standpoint, these data provide evidence about which tests and components are better suited for men compared to women. Men and women are often assigned nearly identical prescription for CFO tests, with the only notable difference being in resistance training loads. The present data not only demonstrate sex differences in performing resistance training exercises despite scaled loads, they also demonstrate differences in several unscaled components. If the decision to scale (or not scale) CFO exercise prescription was intended to equate workloads, the methods employed have not been adequate and this has led to the factors that distinguished top 10% performances to vary between sex divisions. It is thus more prudent to assume that training for the same competition and/or tests should differ between men and women. Researchers interested in validating some of these questions now possess evidence to guide more deliberate and true experimental designs.

## Figures and Tables

**Table 1:** Sample characteristics and competition experience.

	Top 10%	Remaining	
Age (years)			
Men	27.5 ± 4.4	31.2 ± 5.3	#
Women *	30.3 ± 5.4	32.6 ± 7.5	
Height (cm)			
Men	177 ± 7	176 ± 6	
Women *	163 ± 5	163 ± 4	
Weight (kg)			
Men	87.3 ± 9.2	83.6 ± 7.0	#
Women *	61.9 ± 4.6	59.4 ± 5.4	
Consecutive appearances leading up to 2020			
Men	2.8 ± 2.7	1.8 ± 2.1	#
Women	2.9 ± 2.1	1.3 ± 1.7	
Highest CFO Rank			
Men	479 ± 766	9366 ± 10,567	#
Women *	4335 ± 8650	15,234 ± 18,073	
2019 Rank			
Men	694 ± 873	10,749 ± 13,939	#
Women	4177 ± 9186	22,613 ± 37,236	
2020 Rank			
Men	431 ± 290	4854 ± 2411	#
Women *	1959 ± 1308	7106 ± 1706	

* = Significantly (*p* < 0.05) different from men; # = Significantly (*p* < 0.05) different from top 10%.

**Table 2:** Programming and video analysis standards for 2020 CFO tests.

Test 1
15-min time limit to complete 10 sets:
8 × Ground-to-overheads (G2OH; 95 lbs./65 lbs.) * Timing started when barbell plates left floor and ended when both arms reached full, overhead extension on the final repetition of a set.
10 × Bar-facing burpees (BFB) * Timing started when the athlete initiated movement from a standing position towards the floor and ended when both feet landed on the floor after jumping over barbell on the final repetition of a set.
Official Score: TTC or repetitions completed at time limit
Test 2
20-min AMRAP of:
4 × Dumbbell thrusters (DBT; 50 lbs./35 lbs.) * Timing started when the athlete initiated downward movement into the squatting motion while holding dumbbells and ended when both arms reached full, overhead extension on the final repetition of a set.
6 × Toes-to-bar (TTB) * Timing started when the athlete initially left their ground support and were supporting their body weight from the pull-up bar, and ended when their toes were in contact with the bar on the final repetition of a set.
24 × Double-unders (DU) Timing started when the athlete’s feet left the floor, regardless of whether the first repetition was a single-under or DU. DUs counted when double wrist rotations were visible. Timing ended when feet landed on the final repetition of a set.
Official Score: Repetitions completed in 20 min
Test 3
9-min time limit to complete,
21-15-9 repetitions of:
Deadlifts (DL; 225 lbs./155 lbs.) * Timing started when barbell plates left floor and ended when the hips and knees reached full extension on the final repetition of a set.
Handstand push-ups (HSPU) Timing started when the athlete assumed an upside down, handstand position and ended when both arms reached full extension on the final repetition of a set.
Then, 21-15-9 repetitions of:
Deadlifts (315 lbs./205 lbs.) See above.
50′ Handstand walking (HSW’) Timing started when the athlete assumed an upside down, handstand position and ended whenever the athlete began lowering themselves after traveling distances in multiples of 5′ up to 25′.
Official Score: TTC or repetitions completed at time limit
Test 4
20-min time limit to complete:
Alternate the following two exercises:
30 × Box jumps (BJ; 24″/20″) * Timing started when the athlete’s feet left the floor and ended when the athlete stood with hips and knees at full extension atop the box on the final repetition of a set.
Clean and jerks: 15 × (CNJ; 95 lbs./65 lbs.) → 15 × (135 lbs./85 lbs.) → 10 × (185 lbs./115 lbs.) Timing started when barbell plates left floor and ended when both arms reached full, overhead extension on the final repetition of a set.
Then, alternate the following two exercises:
30 × Single-leg squats (SLSQ) Timing started when the athlete initiated downward movement into the squatting motion and ended when the exercising leg and hip reached full extension on the final repetition of a set.
Clean and jerks: 10 × (225 lbs./145 lbs.) → 5 × (275 lbs./175 lbs.) → 5 × (315 lbs./205 lbs.) See above.
Official Score: TTC or repetitions completed at time limit
Test 5
20-min time limit to complete the following in any partitioning order:
40 × Ring muscle-ups (RMU) Timing started when the athlete initially left their ground support and were supporting their body weight from the rings, and ended when both arms reached full, extension atop the rings on the final repetition of a set.
* 80-calorie Rowing (ROW) Timing started when the athlete began pulling on the handle of the rowing ergometer and ended when the handle had reached its furthers point (i.e., closest to the athlete’s chest) on the final pull of a set. Partial strokes were uniformly counted as ½ stroke.
120 × Wall ball shots (WB; 20 lbs./14 lbs. to 10′/9′ target) Timing started when the athlete initiated downward movement into the squatting motion while holding the medicine ball and ended when the ball reached the height of the target on the final repetition of a set.
Official Score: TTC or repetitions completed at time limit

* = Note: Most video submissions did not make the rowing ergometer’s screen clearly visible until 80 calories had been completed. Therefore, the total calories completed within a given period was estimated from the number of strokes completed; a process that assumes that the strength of each rowing stroke was consistent.

**Table 3:** Sex and rank differences in variability of pacing strategy for test 1 (mean ± SD).

	Slope (Per Round)						Coefficient of Variation (%)						
	All Competitors		Top 10%		Remaining		All			Top 10%		Remaining	
	First Half	Last Half	First Half	Last Half	First Half	Last Half	First Half	Last Half		First Half	Last Half	First Half	Last Half
Ground-to-Overheads													
Rate (repetitions·s^−1^)													
Men	−0.01 ± 0.01	0.00 ± 0.02	−0.01 ± 0.01	0.00 ± 0.02	−0.01 ± 0.01	0.00 ± 0.02	7.8 ± 5.8	9.6 ± 7.5	*	5.2 ± 3.0	6.3 ± 4.5	8.5 ± 6.2	10.5 ± 8.0
Women	−0.01 ± 0.02	0.00 ± 0.02	−0.01 ± 0.02	0.00 ± 0.02	−0.01 ± 0.02	0.00 ± 0.02	8.9 ± 7.4	11.0 ± 8.9		7.7 ± 7.2	9.0 ± 6.9	11.0 ± 7.5	14.0 ± 10.8
All	−0.01 ± 0.02	0.00 ± 0.02 †	−0.01 ± 0.02	0.00 ± 0.02	−0.01 ± 0.02	0.00 ± 0.02	8.4 ± 6.7	10.3 ± 8.3 †		7.0 ± 6.5	8.3 ± 6.4	9.3 ± 6.7	11.6 ± 9.1
Breaks (s)										#			
Men	0.35 ± 0.72	−0.05 ± 0.94	0.06 ± 0.23	−0.06 ± 0.70	0.42 ± 0.78	−0.04 ± 1.00	26.6 ± 59.3	52.4 ± 68.0	*	2.2 ± 8.8	26.0 ± 56.0	33.1 ± 65.1	59.4 ± 69.6
Women	0.32 ± 0.77	0.01 ± 0.98	0.21 ± 0.67	0.02 ± 0.70	0.5.0 ± 0.89	−0.02 ± 1.31	27.7 ± 60.0	76.5 ± 84.5		16.4 ± 47.6	78.2 ± 92.0	45.2 ± 72.8	73.7 ± 72.8
All	0.33 ± 0.74	−0.02 ± 0.96 †	0.17 ± 0.59	0.00 ± 0.70	0.45 ± 0.81	−0.04 ± 1.10	27.1 ± 59.4	64.3 ± 77.3 †		12.7 ± 41.5	64.5 ± 86.8	37.1 ± 67.6	64.1 ± 70.6
Transitions to Bar-facing Burpees (s)										#			
Men	0.32 ± 0.38	−0.21 ± 0.42 †	0.24 ± 0.23	0.08 ± 0.31	0.35 ± 0.41	−0.29 ± 0.42	27.4 ± 13.1	30.9 ± 12.4		25.8 ± 14.0	31.3 ± 13.4	27.9 ± 12.9	30.7 ± 12.2
Women	0.43 ± 0.42	−0.35 ± 0.46 *†	0.34 ± 0.40	−0.24 ± 0.36	0.57 ± 0.43	−0.51 ± 0.55	27.6 ± 12.8	28.8 ± 11.8		26.9 ± 14.3	26.9 ± 9.9	28.7 ± 10.4	31.7 ± 14.0
All	0.38 ± 0.40	−0.28 ± 0.44	0.32 ± 0.37	−0.16 ± 0.37 †	0.42 ± 0.43	−0.36 ± 0.47 #†	27.5 ± 12.9	29.8 ± 12.1		26.6 ± 14.1	28.1 ± 11.0	28.1 ± 12.1	31.1 ± 12.7
Bar-facing Burpees													
Rate (repetitions·s^−1^)													
Men	−0.02 ± 0.01	0.03 ± 0.02 †	−0.02 ± 0.01	0.03 ± 0.01	−0.02 ± 0.01	0.03 ± 0.02	12.5 ± 6.5	26.1 ± 10.6 †		11.5 ± 6.5	25.8 ± 11.4	12.7 ± 6.5	26.1 ± 10.4
Women	−0.02 ± 0.01	0.02 ± 0.01 *†	−0.02 ± 0.01	0.02 ± 0.01	−0.02 ± 0.01	0.02 ± 0.02	14.2 ± 6.8	20.1 ± 10.9 *†		13.4 ± 6.7	20.0 ± 9.7	15.4 ± 6.9	20.1 ± 12.8
All	−0.02 ± 0.01	0.02 ± 0.02	−0.02 ± 0.01	0.02 ± 0.01	−0.02 ± 0.01	0.02 ± 0.02	13.3 ± 6.7	23.1 ± 11.1		12.9 ± 6.7	21.6 ± 10.4	13.6 ± 6.7	24.2 ± 11.5
Breaks (s)													
Men	0.01 ± 0.06	−0.04 ± 0.21	0 ± 0	−0.01 ± 0.10	0.02 ± 0.06	−0.04 ± 0.23	13.6 ± 52.2	57.5 ± 89.9		0 ± 0	33.5 ± 66.8	17.2 ± 58.4	63.9 ± 94.6
Women	0.04 ± 0.16	−0.02 ± 0.09	0.01 ± 0.06	−0.02 ± 0.09	0.08 ± 0.23	−0.04 ± 0.09	16.0 ± 55.2	39.2 ± 81.3		9.9 ± 46.6	49.6 ± 90.9	25.3 ± 66.2	23.0 ± 61.7
All	0.02 ± 0.12	−0.03 ± 0.16 †	0.01 ± 0.05	−0.01 ± 0.09	0.04 ± 0.14	−0.04 ± 0.19	14.8 ± 53.5	48.5 ± 86.0 †		7.3 ± 40.1	45.4 ± 85.0	19.8 ± 60.8	50.6 ± 87.1
Transitions to Ground-to-Overheads (s)													
Men	1.15 ± 1.17	−0.97 ± 1.44 †	0.74 ± 0.59	−0.54 ± 1.14	1.26 ± 1.26	−1.09 ± 1.49	31.2 ± 14.6	25.2 ± 13.6	*	28.6 ± 10.9	20.5 ± 13.7	31.8 ± 15.5	26.5 ± 13.4
Women	1.42 ± 1.34	−1.55 ± 2.03 *†	0.94 ± 1.05	−0.93 ± 1.61	2.16 ± 1.43	−2.50 ± 2.27	33.6 ± 16.3	30.7 ± 15.4		31.3 ± 17.1	29.3 ± 14.6	37.3 ± 14.5	32.9 ± 16.5
All	1.29 ± 1.26	−1.26 ± 1.77	0.89 ± 0.95	−0.83 ± 1.50 †	1.56 ± 1.37 #	−1.55 ± 1.89 #†	32.4 ± 15.5	27.9 ± 14.7 †		30.6 ± 15.7	27.0 ± 14.8	33.6 ± 15.3	28.5 ± 14.7
										#			

* = significant (*p* < 0.05) difference between sexes; # = significant (*p* < 0.05) difference between ranks; † = significant (*p* < 0.05) difference between halves.

**Table 4:** Sex and rank differences in variability of pacing strategy for test 2 (mean ± SD).

	Slope (Per Minute)								Coefficient of Variation						
	All Competitors			Top 10%		Remaining			All Competitors			TOP 10%		Remaining	
	First Half	Last Half		First Half	Last Half	First Half	Last Half		First Half	Last Half		First Half	Last Half	First Half	Last Half
Dumbbell Thrusters															
Rate (repetitions·s^−1^)															
Men	0.00 ± 0.01	0.00 ± 0.01		0.00 ± 0.01	0.00 ± 0.01	0.00 ± 0.01	0.00 ± 0.01		8.0 ± 4.2	9.7 ± 6.3	*	7.0 ± 1.3	7.1 ± 1.8	8.2 ± 4.6	10.4 ± 6.8
Women	0.00 ± 0.01	0.00 ± 0.01		0.00 ± 0.01	0.00 ± 0.01	0.00 ± 0.01	0.00 ± 0.02		11.6 ± 6.1	16.4 ± 15.4		10.9 ± 5.0	14.3 ± 9.3	12.6 ± 7.4	19.5 ± 21.2
All	0.00 ± 0.01	0.00 ± 0.01		0.00 ± 0.01	0.00 ± 0.01	0.00 ± 0.01	0.00 ± 0.01		9.8 ± 5.5	13.0 ± 12.1†		9.9 ± 4.6	12.4 ± 8.6	9.7 ± 6.0	13.4 ± 14.0
Breaks (s)												#			
Men	0 ± 0	0.03 ± 0.11		0 ± 0	0.03 ± 0.10	0 ± 0	0.03 ± 0.11		0 ± 0	22.6 ± 79.0 †		0 ± 0	35.3 ± 96.7	0 ± 0	19.4 ± 74.3
Women	0 ± 0	0 ± 0		0 ± 0	0 ± 0	0 ± 0	0.00 ± 0.01		0 ± 0	3.9 ± 34.2 *		0 ± 0	0 ± 0	0 ± 0	9.7 ± 53.9
All	0 ± 0	0.01 ± 0.08		0 ± 0	0.01 ± 0.05	0 ± 0	0.02 ± 0.09		0 ± 0	13.3 ± 61.5		0 ± 0	9.1 ± 50.4	0 ± 0	16.1 ± 68.0
Transitions to toes-to-bar (s)															
Men	0.25 ± 0.24	−0.00 ± 0.33 †		0.14 ± 0.12	−0.12 ± 0.18	0.27 ± 0.26	−0.22 ± 0.35		23.1 ± 8.9	29.9 ± 11.1	*	17.7 ± 3.9	22.3 ± 7.9	24.4 ± 9.3	31.9 ± 11.0
Women	0.40 ± 0.28 *	−0.13 ± 0.30 *†		0.32 ± 0.23	−0.13 ± 0.28	0.51 ± 0.30	−0.13 ± 0.34		33.4 ± 11.6	35.7 ± 12.4		30.5 ± 9.9	32.8 ± 10.9	37.6 ± 12.7	40.0 ± 13.4
All	0.32 ± 0.27	−0.17 ± 0.32		0.27 ± 0.22	−0.13 ± 0.26 †	0.35 ± 0.30 #	−0.19 ± 0.35 †		28.2 ± 11.5	32.8 ± 12.1 †		27.2 ± 10.4	30.1 ± 11.1	28.8 ± 12.2	34.6 ± 12.4
Toes-to-bar												#			
Rate (repetitions·s^−1^)															
Men	0.00 ± 0.01	0.00 ± 0.01	*	0 ± 0	0.00 ± 0.01	0.00 ± 0.01	0.00 ± 0.01		5.6 ± 2.4	7.5 ± 6.2	*	4.6 ± 1.3	4.6 ± 1.4	5.9 ± 2.5	8.2 ± 6.8
Women	0.00 ± 0.01	0.00 ± 0.01		0.00 ± 0.01	0.00 ± 0.01	−0.01 ± 0.02	0.00 ± 0.01		11.4 ± 7.7	14.8 ± 8.1		9.8 ± 4.8	13.7 ± 7.5	13.8 ± 10.2	16.4 ± 8.7
All	0.00 ± 0.01	0.00 ± 0.01		0.00 ± 0.01	0.00 ± 0.01	0.00 ± 0.01	0.00 ± 0.01		8.5 ± 6.3	11.1 ± 8.1 †		8.5 ± 4.8	11.3 ± 7.6	8.5 ± 7.2	10.9 ± 8.4
Breaks (s)												#			
Men	0.01 ± 0.06	0.06 ± 0.19		0 ± 0	0.05 ± 0.12	0.02 ± 0.07	0.06 ± 0.21		9.1 ± 42.0	57.0 ± 104.3		0 ± 0	41.0 ± 102.1	11.5 ± 46.9	61.1 ± 105.3
Women	0.05 ± 0.15	0.00 ± 0.17		0.05 ± 0.16	−0.01 ± 0.14	0.04 ± 0.14	0.02 ± 0.21		14.8 ± 50.1	38.8 ± 85.1		7.4 ± 30.7	33.6 ± 86.1	25.6 ± 68.8	46.5 ± 84.5
All	0.03 ± 0.11	0.03 ± 0.18		0.04 ± 0.14	0.01 ± 0.14	0.03 ± 0.10	0.05 ± 0.21		11.9 ± 46.1	47.9 ± 95.4 †		5.5 ± 26.6	35.5 ± 89.6	16.2 ± 55.2	56.2 ± 98.6
Transitions to Double-unders (s)															
Men	0.31 ± 0.24	−0.10 ± 0.29 †		0.31 ± 0.27	−0.04 ± 0.18	0.31 ± 0.24	−0.11 ± 0.31		23.0 ± 10.4	26.9 ± 10.9		23.9 ± 14.1	17.5 ± 6.5 bcd	22.7 ± 9.4 d	29.3 ± 10.5 ab†
Women	0.30 ± 0.29	−0.24 ± 0.33 *†		0.31 ± 0.24	−0.20 ± 0.28	0.29 ± 0.36	−0.30 ± 0.38		29.0 ± 9.1	34.2 ± 10.3		26.8 ± 8.2	32.6 ± 10.4 a†	31.8 ± 9.0 c	36.8 ± 9.8 ac
All	0.30 ± 0.27	−0.17 ± 0.31		0.31 ± 0.25	−0.16 ± 0.26	0.30 ± 0.28	−0.18 ± 0.34		26.0 ± 10.2	30.5 ± 11.2		26.0 ± 10.0	28.7 ± 11.6	26.0 ± 10.4	31.7 ± 10.8
Double-unders															
Rate (repetitions·s^−1^)															
Men	0.00 ± 0.01	0.00 ± 0.02		0.00 ± 0.01	0.00 ± 0.01	0.00 ± 0.01	−0.01 ± 0.02	*	5.1 ± 2.9	7.8 ± 6.7	*	3.9 ± 1.6	4.6 ± 1.4	5.4 ± 3.1	8.7 ± 7.3
Women	0.00 ± 0.02	0.00 ± 0.03		0.00 ± 0.02	0.00 ± 0.02	0.01 ± 0.03	0.00 ± 0.03		12.7 ± 7.8	14.5 ± 8.7		10.2 ± 5.0	12.1 ± 6.3	16.4 ± 9.7	18.2 ± 10.4
All	0.00 ± 0.02	0.00 ± 0.02		0.00 ± 0.02	0.00 ± 0.02	0.00 ± 0.02	0.00 ± 0.03		8.9 ± 7.0	11.2 ± 8.4 †		8.6 ± 5.2	10.2 ± 6.4	9.0 ± 8.0	11.8 ± 9.5
Breaks (s)												#			
Men	0.03 ± 0.14	0.06 ± 0.19		0.01 ± 0.12	0.00 ± 0.18	0.04 ± 0.14	0.08 ± 0.20		149.4 ± 105.0	166.6 ± 97.0	*	146.4 ± 117.0	206.2 ± 91.7	150.1 ± 102.7	156.4 ± 96.4
Women	0.05 ± 0.13	0.04 ± 0.19		0.04 ± 0.10	0.02 ± 0.18	0.06 ± 0.18	0.06 ± 0.19		57.0 ± 106.9	81.4 ± 113.2		50.5 ± 103.2	83.7 ± 116.4	66.6 ± 113.2	77.9 ± 110.1
All	0.04 ± 0.14	0.05 ± 0.19		0.03 ± 0.10	0.02 ± 0.18	0.05 ± 0.15	0.07 ± 0.19		103.5 ± 115.3	124.3 ± 113.4 †		75.2 ± 114.1	115.3 ± 122.4	122.3 ± 112.9	130.2 ± 107.2
Transitions to Dumbbell Thrusters (s)				#											
Men	0.32 ± 0.50	−0.35 ± 0.48 †		0.17 ± 0.22	−0.13 ± 0.20	0.36 ± 0.54	−0.41 ± 0.52		25.1 ± 9.6	32.5 ± 10.1	*	21.5 ± 8.0	23.9 ± 6.8	26.1 ± 9.8	34.8 ± 9.6
Women	0.52 ± 0.44 *	−0.41 ± 0.42 *†		0.42 ± 0.40	−0.37 ± 0.39	0.68 ± 0.46	−0.49 ± 0.46		33.7 ± 13.2	37.0 ± 11.5		29.4 ± 11.8	33.0 ± 10.1	40.1 ± 12.9	43.4 ± 10.8
All	0.42 ± 0.48	−0.38 ± 0.45		0.36 ± 0.38	−0.31 ± 0.36 †	0.46 ± 0.53 #	−0.43 ± 0.50 †		29.4 ± 12.3	34.7 ± 11.0 †		27.4 ± 11.4	30.6 ± 10.1	30.7 ± 12.7	37.5 ± 10.7
												#			

* = significant (*p* < 0.05) difference between sexes; # = significant (*p* < 0.05) difference between ranks; † = significant (*p* < 0.05) difference between first and last halves; a–b = significantly (*p* < 0.05) different than (a. top 10% men; b. top 10% women; c. remaining men; d. remaining women).

**Table 5:** Sex and rank differences in variability of pacing strategy for test 3 (mean ± SD).

	Slope (Per Round)							Coefficient of Variation (%)						
	All Competitors			Top 10%		Remaining		All Competitors			Top 10%		Remaining	
	First Half	Last Half		First Half	Last Half	First Half	Last Half	First Half	Last Half		First Half	Last Half	First Half	Last Half
Deadlifts														
Rate (repetitions·s^−1^)														
Men	−0.06 ± 0.10	0.06 ± 0.10		−0.05 ± 0.06	0.04 ± 0.05	−0.04 ± 0.08	0.07 ± 0.12	21.1 ± 13.1	14.9 ± 23.6		15.8 ± 6.2	20.1 ± 16.7	22.3 ± 13.9	13.7 ± 24.8
Women	−0.04 ± 0.06	0.10 ± 0.11		−0.06 ± 0.06	−0.06 ± 0.00	0.10 ± 0.12	0.06 ± 0.03	17.8 ± 9.8	10.3 ± 18.9		15.6 ± 9.4	21.1 ± 10.0	16.2 ± 22.1	1.7 ± 6.4
All	−0.05 ± 0.09	0.07 ± 0.11 †		−0.05 ± 0.06	0.08 ± 0.10	−0.05 ± 0.10	0.07 ± 0.11	19.4 ± 11.6	12.7 ± 21.5		15.6 ± 8.6	17.2 ± 20.9	21.8 ± 12.6	9.9 ± 21.5 #†
Breaks (s)														
Men	0.24 ± 2.11	−6.34 ± 7.16		0.02 ± 1.38	−2.55 ± 2.13	0.30 ± 1.90	−8.23 ± 8.03	68.9 ± 54.5	28.1 ± 39.8		70.2 ± 56.3	36.6 ± 29.5	68.6 ± 54.5	26.1 ± 41.8
Women	0.17 ± 2.07	−5.30 ± 2.98		0.26 ± 1.69	0.25 ± 0.35	−5.32 ± 3.07	−5.07 ± 2.38	59.6 ± 54.9	28.1 ± 45.9		67.2 ± 58.6	56.6 ± 48.1	42.8 ± 50.3	6.3 ± 27.1
All	0.21 ± 2.09	−5.93 ± 5.88 †		0.05 ± 1.61	−4.33 ± 3.05	0.31 ± 2.34	−8.02 ± 7.81	64.3 ± 54.7	28.1 ± 42.8 †		66.8 ± 57.8	41.3 ± 45.9	62.7 ± 53.0	19.8 ± 38.7
Transitions to Handstand Push-ups/Handstand Walking (s)											#			
Men	2.59 ± 2.27	−3.10 ± 3.90		1.11 ± 0.74		2.93 ± 2.37		31.4 ± 16.8	10.7 ± 18.0		25.1 ± 13.7		31.3 ± 18.2	
Women	1.91 ± 2.86	−3.73 ± 5.83		1.80 ± 2.43		2.08 ± 3.38		29.9 ± 21.9	9.4 ± 21.0		30.5 ± 18.8		11.6 ± 22.9	
All	2.25 ± 2.59	−3.34 ± 4.66		1.63 ± 2.16	−2.52 ± 4.44	2.64 ± 2.77	−6.00 ± 4.60	30.6 ± 19.4	10.1 ± 19.3		28.3 ± 18.1	14.6 ± 21.3	32.1 ± 20.2	5.7 ± 16.2
Handstand Push-ups/Handstand Walking				#							#			
Rate (repetitions·s^−1^)														
Men	−0.08 ± 0.08	0.02 ± 0.06	*	−0.09 ± 0.06		−0.07 ± 0.08		18.5 ± 15.9	4.6 ± 8.3		18.8 ± 10.3		25.9 ± 14.4	
Women	−0.05 ± 0.05	0.00 ± 0.09		−0.06 ± 0.06		−0.03 ± 0.03		15.6 ± 16.5	11.1 ± 30.6		26.1 ± 14.7		13.8 ± 33.7	
All	−0.06 ± 0.07	0.01 ± 0.07		−0.07 ± 0.06	0.01 ± 0.07	−0.06 ± 0.07	0.01 ± 0.10	17.0 ± 16.2	7.5 ± 21.4		21.2 ± 15.3	12.4 ± 28.8	14.5 ± 16.3	2.7 ± 8.0
Breaks (s)														
Men	−0.03 ± 2.80	−3.64 ± 4.56		−0.18 ± 1.44		0.00 ± 3.04		37.0 ± 53.4	16.9 ± 29.4		48.4 ± 73.3		48.4 ± 51.0	
Women	−0.06 ± 2.57	−4.30 ± 4.27		0.31 ± 2.05		−0.57 ± 3.12		21.4 ± 39.2	18.8 ± 37.0		36.1 ± 49.0		23.4 ± 40.1	
All	−0.04 ± 2.68	−3.9 ± 4.39		0.19 ± 1.92	−3.23 ± 3.55	−0.19 ± 3.06	−6.06 ± 6.25	29.2 ± 47.3	17.7 ± 32.8		34.7 ± 54.2	25.5 ± 34.7	25.8 ± 42.5	10.2 ± 29.3
Transitions to Deadlifts (s)														
Men	6.97 ± 4.20	−4.60 ± 6.71	*	6.68 ± 3.55		7.04 ± 4.35		34.2 ± 26.0	21.0 ± 21.5	*	51.0 ± 15.9		30.5 ± 26.5	
Women	4.17 ± 5.22	−5.00 ± 3.56		4.35 ± 4.58		3.90 ± 6.11		25.3 ± 27.4	27.4 ± 16.8		37.2 ± 27.5		8.9 ± 17.1	
All	5.59 ± 4.92	−4.73 ± 5.80		4.91 ± 4.44	−4.50 ± 6.04	6.02 ± 5.17	−7.00 ± 1.41	29.8 ± 27.0	23.1 ± 19.9		40.5 ± 25.7	22.2 ± 20.7	23.1 ± 25.7	31.8 ± 6.4
											#			

* = Significant (*p* < 0.05) difference between sexes; # = Significant (*p* < 0.05) difference between ranks; † = Significant (*p* < 0.05) difference between first and last halves.

**Table 6:** Sex and rank differences in variability of pacing strategy for test 4 (mean ± SD).

	Slope (per round)							Coefficient of Variation (%)						
	All Competitors			Top 10%		Remaining		All Competitors			Top 10%		Remaining	
	First Half	Last Half		First Half	Last Half	First Half	Last Half	First Half	Last Half		First Half	Last Half	First Half	Last Half
Box Jumps–Single-leg Squats														
Rate (repetitions·s^−1^)														
Men	−0.05 ± 0.05	0.03 ± 0.07	*	−0.06 ± 0.03	−0.01 ± 0.03	−0.05 ± 0.05	0.04 ± 0.08	15.6 ± 8.7	7.0 ± 11.1		14.1 ± 6.5	8.5 ± 3.1	16.0 ± 9.2	6.6 ± 12.4
Women	−0.05 ± 0.03	0.04 ± 0.08		−0.05 ± 0.03	0.04 ± 0.07	−0.06 ± 0.03	0.05 ± 0.10	13.5 ± 6.9	6.1 ± 10.2		13.0 ± 7.0	9.3 ± 11.1	14.5 ± 6.7	1.1 ± 5.9
All	−0.05 ± 0.04	0.03 ± 0.08 †		−0.05 ± 0.03	−0.05 ± 0.05	0.03 ± 0.07	0.04 ± 0.08	14.5 ± 7.9	6.6 ± 10.7		13.3 ± 6.8	15.4 ± 8.4	9.1 ± 9.7	4.7 ± 11.0 #†
Breaks (s)														
Men	0.27 ± 0.70	−0.88 ± 1.90		0.31 ± 0.96	0.57 ± 0.88	0.27 ± 0.63	−1.25 ± 1.92	22.8 ± 54.1	89.5 ± 65.5		21.7 ± 59.2	73.6 ± 76.7	23.1 ± 53.2	93.8 ± 62
Women	0.20 ± 0.59	−1.04 ± 1.42 *†		0.21 ± 0.58	−0.64 ± 1.13	0.19 ± 0.60	−1.65 ± 1.61	16.1 ± 46.6	82.1 ± 70.9		18.6 ± 51.1	76.4 ± 69.3	12.1 ± 38.8	91.0 ± 73.7
All	0.24 ± 0.65	−0.96 ± 1.68		0.24 ± 0.69	0.24 ± 0.62	−0.33 ± 1.19	−1.38 ± 1.83 #†	19.4 ± 50.4	85.7 ± 68.2 †		19.4 ± 52.8	19.4 ± 48.8	75.7 ± 70.6	92.8 ± 65.8
Transitions to Clean and Jerks (s)														
Men	6.32 ± 5.39	31.42 ± 33.54		2.06 ± 1.58	23.53 ± 24.44	6.87 ± 5.49	33.85 ± 35.73	53.5 ± 27.2	22.1 ± 34.3		34.6 ± 18.4 cd	63.6 ± 27.5 bcd†	58.8 ± 27.1 a	10.7 ± 26.2 abd†
Women	7.15 ± 7.70	24.41 ± 45.15		5.01 ± 6.24	23.42 ± 36.02	7.18 ± 3.62	27.06 ± 65.13	58.4 ± 28.8	17.7 ± 29.9		53.2 ± 31.6	29.1 ± 33.8 acd†	66.6 ± 21.6 a	0.1 ± 0.1 abc†
All	6.73 ± 6.63	28.05 ± 39.55 †		4.41 ± 5.68	8.27 ± 6.79	23.45 ± 33.22	32.17 ± 44.32	56.0 ± 28.0	19.9 ± 32.1		48.5 ± 29.9	61.4 ± 25.5	37.8 ± 35.5	7.0 ± 21.8
Clean and Jerks														
Rate (repetitions·s^−1^)														
Men	−0.09 ± 0.02	−0.04 ± 0.01		−0.09 ± 0.02	−0.03 ± 0.01	−0.09 ± 0.03	−0.04 ± 0.01	48 ± 10.6	28.1 ± 41.9		43.8 ± 9.4	78.8 ± 24.4 bcd†	49.2 ± 10.7	14.1 ± 34.2 ab†
Women	−0.09 ± 0.02	−0.04 ± 0.01		−0.09 ± 0.02	−0.04 ± 0.01	−0.10 ± 0.03	−0.03 ± 0.01	48.5 ± 11.6	25.8 ± 41.9		43.7 ± 10.2	42.3 ± 46.8 acd	56.2 ± 9.5	0.1 ± 0.1 ab†
All	−0.09 ± 0.02	−0.04 ± 0.01 †		−0.09 ± 0.02	−0.09 ± 0.03	−0.04 ± 0.01	−0.04 ± 0.01	48.3 ± 11.1	26.9 ± 41.8		43.7 ± 10.0	51.5 ± 10.8	51.6 ± 45	9.3 ± 28.5
Breaks (s)														
Men	2.07 ± 2.91	−5.22 ± 12.92		0.73 ± 0.91	12.93 ± 13.91 bcd†	2.41 ± 3.14	−9.83 ± 7.51 abd†	71.3 ± 44.5	122.5 ± 38.1	*	78.0 ± 54.2	88.1 ± 22.0	69.5 ± 41.9	132.0 ± 36.2
Women	1.89 ± 1.92	−7.65 ± 10.49		1.43 ± 1.61	−2.72 ± 8.6 acd	2.58 ± 2.17	−15.11 ± 8.58 abc†	83.8 ± 47.7	119.7 ± 46.8		83.5 ± 47.7	101.6 ± 40.7	84.2 ± 48.6	147.9 ± 41.8
All	1.98 ± 2.46	−6.42 ± 11.80		1.26 ± 1.49	2.47 ± 2.85	1.25 ± 12.2	−11.57 ± 8.22	77.7 ± 46.5	121.1 ± 42.6		82.1 ± 49.0	74.5 ± 44.5 †	98.2 ± 37.2	137.4 ± 38.7 #†
Transitions to Box Jumps–Single-leg Squats (s)														
Men	5.48 ± 7.74	−3.16 ± 4.17		0.91 ± 1.82	−1.38 ± 1.82	5.97 ± 7.17	−4.94 ± 5.09	58.9 ± 35.5	14.9 ± 28.1		41.3 ± 23.7	15 ± 17.5	63.8 ± 36.8	14.9 ± 30.6
Women	3.64 ± 5.93	−3.10 ± 4.89		2.52 ± 2.85	−3.21 ± 4.94	1.50 ± 0.00	0.00 ± 0.00	51 ± 34.2	15.5 ± 28.4		51.3 ± 35.1	25.3 ± 32.9	50.5 ± 33.2	0.1 ± 0.1 *
All	4.57 ± 6.94	−3.13 ± 4.49 †		2.48 ± 3.48	5.95 ± 8.22	−2.55 ± 4.16	−4.65 ± 5.07	54.9 ± 35	15.2 ± 28.2		48.8 ± 32.7	59.3 ± 36.0 †	22.7 ± 29.9	9.8 ± 25.7 †

* = sgnificant (*p* < 0.05) difference between sexes; # = significant (*p* < 0.05) difference between ranks; † = Significant (*p* < 0.05) difference between first and last halves; a–b = significantly (*p* < 0.05) different than (a. top 10% men; b. top 10% women; c. remaining men; d. remaining women).

**Table 7:** Sex and rank differences in variability of pacing strategy for test 5 (mean ± SD).

	Slope (Per Minute)				Coefficient of Variation (%)			
	All Competitors		Top 10%	Remaining	All Competitors		Top 10%	Remaining
Ring Muscle-ups								
Rate (repetitions × s^−1^)								
Men	−0.01 ± 0.01		−0.01 ± 0.01	−0.01 ± 0.01	12.7 ± 10.1	*	9.1 ± 5.0	13.6 ± 10.8
Women	−0.01 ± 0.01		−0.01 ± 0.01	−0.01 ± 0.01	20.1 ± 9.4		18.2 ± 8.5	24.3 ± 9.9
All	−0.01 ± 0.01		−0.01 ± 0.01	−0.01 ± 0.01	16.3 ± 10.4		16.0 ± 8.7	16.5 ± 11.6
Breaks (n)							#	
Men	0.02 ± 0.04	*	0.01 ± 0.02	0.02 ± 0.04	240.4 ± 102.8	*	323.3 ± 22.5	227.1 ± 104.4
Women	0.09 ± 0.10		0.08 ± 0.07	0.12 ± 0.13	128.8 ± 90.7		152.2 ± 87.0	97.9 ± 86.2
All	0.06 ± 0.08		0.06 ± 0.06	0.06 ± 0.09	163.2 ± 107.7		168.8 ± 97.3	158.9 ± 115
Break Time (s)			#				#	
Men	0.22 ± 0.54		0.07 ± 0.13 bd	0.26 ± 0.59 bd	241.2 ± 99.9	*	323.3 ± 22.5	227.6 ± 101.3
Women	1.36 ± 1.39		0.97 ± 0.99 acd	2.00 ± 1.70 abc	128.8 ± 90.1		150.9 ± 85.6	99.6 ± 87.5
All	0.79 ± 1.20		0.75 ± 0.94	0.82 ± 1.35	162.7 ± 106.5		167.7 ± 96.3	158.7 ± 113.7
Rowing							#	
Calories per stroke								
Men	0.00 ± 0.01	*	0.00 ± 0.01	0.00 ± 0.01	11.6 ± 8.0		8.0 ± 5.5	12.6 ± 8.3 d
Women	0.00 ± 0.01		−0.01 ± 0.01	0.00 ± 0.01	9.9 ± 6.2		11.2 ± 6.7	7.9 ± 4.8 c
All	0.00 ± 0.01		0.00 ± 0.01	0.00 ± 0.01	10.7 ± 7.2		10.4 ± 6.6	10.9 ± 7.6
Breaks (n)								
Men	0.00 ± 0.00		0.00 ± 0.00	0.00 ± 0.00	1.0 ± 8.9		0.1 ± 0.1	1.3 ± 10.0
Women	−0.01 ± 0.01		−0.01 ± 0.01	0.00 ± 0.00	8.4 ± 52.1		14.1 ± 66.9	0.1 ± 0.1
All	−0.01 ± 0.01		−0.01 ± 0.01	0.00 ± 0.00	4.8 ± 38.1		10.7 ± 58.6	0.9 ± 8.1
Break Time (s)								
Men	0.00 ± 0.02		0.00 ± 0.01	0.01 ± 0.02	1.3 ± 10.9		0.1 ± 0.1	1.6 ± 12.2
Women	−0.01 ± 0.01		−0.01 ± 0.01	0.00 ± 0.01	8.4 ± 52.1		14.1 ± 66.9	0.1 ± 0.1
All	0.00 ± 0.01		−0.01 ± 0.01	0.00 ± 0.02	4.9 ± 38.4		10.7 ± 58.6	1.0 ± 9.8
Wall Ball Shots								
Rate (repetitions × s^−1^)								
Men	0.01 ± 0.01		0.01 ± 0.01	0.01 ± 0.01	9.7 ± 9.0		7.9 ± 8.6	10.1 ± 9.1
Women	0.01 ± 0.01		0.01 ± 0.01	0.01 ± 0.01	11.4 ± 9.8		11.8 ± 9.4	10.8 ± 10.3
All	0.01 ± 0.01		0.01 ± 0.01	0.01 ± 0.01	10.6 ± 9.5		10.9 ± 9.4	10.3 ± 9.5
Breaks (n)								
Men	0.01 ± 0.05	*	0.00 ± 0.01	0.01 ± 0.06	60.8 ± 104.6	*	11.4 ± 42.7	73.3 ± 111.7
Women	0.03 ± 0.06		0.03 ± 0.05	0.03 ± 0.06	95.7 ± 108.6		99.3 ± 115.6	90.6 ± 97.2
All	0.02 ± 0.05		0.02 ± 0.04	0.02 ± 0.06	78.8 ± 108.1		78.1 ± 109.5	79.4 ± 107.1
Break Time (s)								
Men	0.12 ± 0.30	*	0.01 ± 0.02	0.15 ± 0.33	62.5 ± 106.2	*	12.0 ± 44.8	75.3 ± 113.2
Women	0.29 ± 0.48		0.23 ± 0.37	0.39 ± 0.59	102.2 ± 111.7		105.0 ± 118.8	98.2 ± 100.3
All	0.21 ± 0.41		0.18 ± 0.34	0.24 ± 0.46	83.0 ± 110.9		82.5 ± 113	83.3 ± 109.4
Transitions (n)			#					
Men	−0.06 ± 0.08		−0.08 ± 0.10	−0.06 ± 0.07	51.4 ± 20.4		55.5 ± 15.9 d	50.3 ± 21.3 d
Women	−0.05 ± 0.05		−0.05 ± 0.05	−0.05 ± 0.05	69.5 ± 34.2		62.1 ± 25.6 d	80.4 ± 41.5 abc
All	−0.06 ± 0.07		−0.06 ± 0.07	−0.06 ± 0.07	60.7 ± 29.8		60.5 ± 23.8	60.9 ± 33.3
Transition Time (s)								
Men	0.43 ± 0.48		0.73 ± 0.60 d	0.36 ± 0.41 d	41.8 ± 13.6		44.5 ± 11.9	41.1 ± 14.0 d
Women	0.97 ± 1.18		0.51 ± 0.53 d	1.65 ± 1.51 abc	53.8 ± 24.8		49.2 ± 22.8	60.6 ± 26.1 c
All	0.71 ± 0.95		0.56 ± 0.56	0.81 ± 1.14	48.0 ± 21.1		48.0 ± 20.8	47.9 ± 21.3

* = significant (*p* < 0.05) difference between sexes; # = significant (*p* < 0.05) difference between ranks; a–b = significantly (*p* < 0.05) different than (a. top 10% men; b. top 10% women; c. remaining men; d. remaining women).

## Data Availability

All data are publicly available at: https://games.crossfit.com/leaderboard/open/ (accessed on 11 October 2019).

## References

[1] CrossFit (2022). Finding the Fittest on Earth. CrossFit Games.

[2] CrossFit (2021). Open Workouts. CrossFit Games.

[3] CrossFit (2022). Games Competition Rulebook. CrossFit J..

[4] CrossFit Leaderboard. http://games.crossfit.com/leaderboard.

[5] Mangine G.T., Grundlingh N., Feito Y. (2023). Differential improvements between men and women in repeated CrossFit^®^ Open workouts. medRxiv.

[6] Butcher S.J., Neyedly T.J., Horvey K.J., Benko C.R. (2015). Do physiological measures predict selected CrossFit^®^ benchmark performance?. Open Access J. Sports Med..

[7] Bellar D., Hatchett A., Judge L., Breaux M., Marcus L. (2015). The relationship of aerobic capacity, anaerobic peak power and experience to performance in CrossFit exercise. Biol. Sport.

[8] Feito Y., Giardina M.J., Butcher S., Mangine G.T. (2018). Repeated anaerobic tests predict performance among a group of advanced CrossFit-trained athletes. Appl. Physiol. Nutr. Metab..

[9] Dexheimer J.D., Schroeder E.T., Sawyer B.J., Pettitt R.W., Aguinaldo A.L., Torrence W.A. (2019). Physiological Performance Measures as Indicators of CrossFit^®^ Performance. Sports.

[10] Zeitz E.K., Cook L.F., Dexheimer J.D., Lemez S., Leyva W.D., Terbio I.Y., Tran J.R., Jo E. (2020). The relationship between Crossfit^®^ performance and laboratory-based measurements of fitness. Sports.

[11] Carreker J.D.D., Grosicki G.J. (2020). Physiological predictors of performance on the CrossFit^®^ “Murph” challenge. Sports.

[12] Mangine G.T., Tankersley J.E., McDougle J.M., Velazquez N., Roberts M.D., Esmat T.A., VanDusseldorp T.A., Feito Y. (2020). Predictors of CrossFit Open performance. Sports.

[13] Mangine G.T., Mcdougle J.M., Feito Y. (2022). Relationships Between Body Composition and “Fran” Performance are Modulated by Competition Class and Skill. Front. Physiol..

[14] Mangine G.T., Feito Y., Tankersley J.E., McDougle J.M., Kliszczewicz B.M. (2021). Workout Pacing Predictors of Crossfit Open Performance: A Pilot Study. J. Hum. Kinet..

[15] Mangine G.T., Seay T.R. (2022). Quantifying CrossFit^®^: Potential solutions for monitoring multimodal workloads and identifying training targets. Front. Sports Act. Living.

[16] Glassman G. (2010). The CrossFit training guide. CrossFit J..

[17] Mangine G.T., Grundlingh N., Feito Y. (2023). Normative Scores for CrossFit^®^ Open Workouts: 2011–2022. Sports.

[18] Sandbakk Ø., Solli G.S., Holmberg H.-C. (2018). Sex differences in world-record performance: The influence of sport discipline and competition duration. Int. J. Sports Physiol. Perform..

[19] Hunter S.K. (2016). The relevance of sex differences in performance fatigability. Med. Sci. Sports Exerc..

[20] Huebner M., Perperoglou A. (2020). Sex differences and impact of body mass on performance from childhood to senior athletes in Olympic weightlifting. PLoS ONE.

[21] Bishop D., Edge J., Goodman C. (2004). Muscle buffer capacity and aerobic fitness are associated with repeated-sprint ability in women. Eur. J. Appl. Physiol..

[22] Buckley S., Knapp K., Lackie A., Lewry C., Horvey K., Benko C., Trinh J., Butcher S. (2015). Multimodal high-intensity interval training increases muscle function and metabolic performance in females. Appl. Physiol. Nutr. Metab..

[23] Toledo R., Dias M.R., Toledo R., Erotides R., Pinto D.S., Reis V.M., Novaes J.S., Vianna J.M., Heinrich K.M. (2021). Comparison of Physiological Responses and Training Load between Different CrossFit^®^ Workouts with Equalized Volume in Men and Women. Life.

[24] Mangine G.T., Cebulla B., Feito Y. (2018). Normative values for self-reported benchmark workout scores in CrossFit^®^ practitioners. Sports Med.-Open.

[25] Cohen J. (1988). The effect size index: F. Statistical Power Analysis for the Behavioral Sciences.

[26] Frontera W.R., Hughes V.A., Lutz K.J., Evans W.J. (1991). A cross-sectional study of muscle strength and mass in 45-to 78-yr-old men and women. J. Appl. Physiol..

[27] Doherty T.J. (2001). The influence of aging and sex on skeletal muscle mass and strength. Curr. Opin. Clin. Nutr. Metab. Care.

[28] Bishop P., Cureton K., Collins M. (1987). Sex difference in muscular strength in equally-trained men and women. Ergonomics.

